# pOpsicle: An all-optical reporter system for synaptic vesicle recycling combining pH-sensitive fluorescent proteins with optogenetic manipulation of neuronal activity

**DOI:** 10.3389/fncel.2023.1120651

**Published:** 2023-03-31

**Authors:** Marius Seidenthal, Barbara Jánosi, Nils Rosenkranz, Noah Schuh, Nora Elvers, Miles Willoughby, Xinda Zhao, Alexander Gottschalk

**Affiliations:** ^1^Buchmann Institute for Molecular Life Sciences, Goethe University Frankfurt, Frankfurt, Germany; ^2^Institute of Biophysical Chemistry, Department of Biochemistry, Chemistry and Pharmacy, Goethe University, Frankfurt, Germany

**Keywords:** neurotransmission, exo- and endocytosis, optogenetics, *Caenorhabditis elegans*, synaptic plasticity

## Abstract

pH-sensitive fluorescent proteins are widely used to study synaptic vesicle (SV) fusion and recycling. When targeted to the lumen of SVs, fluorescence of these proteins is quenched by the acidic pH. Following SV fusion, they are exposed to extracellular neutral pH, resulting in a fluorescence increase. SV fusion, recycling and acidification can thus be tracked by tagging integral SV proteins with pH-sensitive proteins. Neurotransmission is generally activated by electrical stimulation, which is not feasible in small, intact animals. Previous *in vivo* approaches depended on distinct (sensory) stimuli, thus limiting the addressable neuron types. To overcome these limitations, we established an all-optical approach to stimulate and visualize SV fusion and recycling. We combined distinct pH-sensitive fluorescent proteins (inserted into the SV protein synaptogyrin) and light-gated channelrhodopsins (ChRs) for optical stimulation, overcoming optical crosstalk and thus enabling an all-optical approach. We generated two different variants of the pH-sensitive optogenetic reporter of vesicle recycling (pOpsicle) and tested them in cholinergic neurons of intact *Caenorhabditis elegans* nematodes. First, we combined the red fluorescent protein pHuji with the blue-light gated ChR2(H134R), and second, the green fluorescent pHluorin combined with the novel red-shifted ChR ChrimsonSA. In both cases, fluorescence increases were observed after optical stimulation. Increase and subsequent decline of fluorescence was affected by mutations of proteins involved in SV fusion and endocytosis. These results establish pOpsicle as a non-invasive, all-optical approach to investigate different steps of the SV cycle.

## 1. Introduction

Chemical synaptic transmission, the release of neurotransmitters into the synaptic cleft, depends on synaptic vesicle (SV) exocytosis (Sudhof, 2013). To efficiently sustain neurotransmitter release during phases of (high) neuronal activity, SV-associated proteins and lipids must be recycled from the plasma membrane, thus allowing regeneration of ready-to-release SVs (Gan and Watanabe, 2018; Chanaday et al., 2019). Several modes of SV recycling have been uncovered, including classical clathrin-mediated endocytosis, activity-dependent bulk endocytosis, kiss-and-run release and the recently described ultrafast endocytosis (Heuser and Reese, 1973; Kittelmann et al., 2013; Watanabe et al., 2013a,b; Morton et al., 2015; Watanabe and Boucrot, 2017; Shin et al., 2021). It is still under debate which of these processes happen under which conditions, and whether some of these represent short-cuts in the SV cycle, e.g., bypassing the endosome. Also, the exact involvement of known, and the discovery of novel, recycling factors mediating these events is the subject of ongoing research (Gan and Watanabe, 2018; Yu et al., 2018). To study SV fusion and recycling, methods such as electron microscopy (EM), measurement of membrane capacitance, and super-resolution microscopy are used. However, these methods are either limited in their temporal resolution (EM) or applicability to different neuronal cell types (membrane capacitance measurements and super-resolution microscopy) (von Gersdorff and Matthews, 1999; Yu et al., 2018; Shin et al., 2021).

**FIGURE 1:**
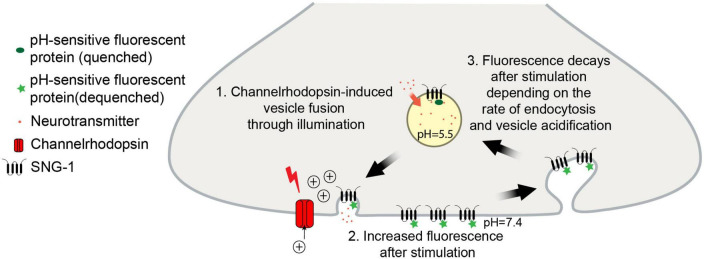
Schematic of the pOpsicle assay. pH-sensitive fluorescent proteins fused to SNG-1 are exposed to the neutral extracellular medium in response to optogenetic stimulation which leads to an increase in fluorescence.

Another method to indirectly visualize and quantify exocytosis and recycling of proteins is through tagging with pH-sensitive fluorescent proteins (Miesenbock et al., 1998). SVs acidification precedes and is required for refilling with neurotransmitters during the recycling process (Egashira et al., 2015; Gowrisankaran and Milosevic, 2020). For this reason, the fluorescence of pH-sensitive proteins, such as the green fluorescent pHluorin, when targeted to the acidic intraluminal side of the SV membrane, is quenched by the low-pH environment (Sankaranarayanan et al., 2000). Typically, the utilized reporters are either fused to the vesicular glutamate transporter or inserted into loops of the tetraspan membrane protein synaptophysin (Voglmaier et al., 2006; Luo et al., 2021). Upon SV fusion with the plasma membrane, the fluorescence increases due to exposure to the neutral extracellular medium and dequenching of the fluorophore (Figure 1). Following stimulation, the fluorescence decreases depending on the rate of endocytosis, sorting and reacidification of SVs. This way, positive or negative influences on the speed of SV retrieval can be quantified (Morton et al., 2015; Watanabe et al., 2018). Several variants of pH-sensitive fluorescent proteins have been created, covering almost the entire spectrum of visible light (Miesenbock et al., 1998; Shen et al., 2014; Liu et al., 2021). This opens the door for multiplexing with other fluorophores or optical actuators (Li and Tsien, 2012; Jackson and Burrone, 2016).

Most studies utilizing pH-sensitive fluorescent proteins in mammalian organisms are performed using cultured neurons or semi-intact preparations such as hippocampal slices (Rose et al., 2013; Watanabe et al., 2018). *In vivo* studies are rare and usually performed in translucent non-mammalian model systems such as *Danio rerio* and *Drosophila melanogaster* larvae or *Caenorhabditis elegans* (Poskanzer et al., 2003; Koudelka et al., 2016; Ventimiglia and Bargmann, 2017; Seitz and Rizzoli, 2019). Electrical stimulation can be used to trigger neurotransmission, but this is comparatively invasive and labor-intensive, particularly if it involves dissection. Alternatively, exposure to stimuli such as odors can be used. However, this is difficult to control, and limited to applications in sensory neurons (Choi et al., 2021). Thus, an all-optical solution that is not limited to certain cell types would be ideal, e.g., a combination of genetically encoded non-invasive tools for *in vivo* stimulation of neurons, and pH-sensitive fluorescent proteins. One possibility to manipulate neurotransmission is through transgenic expression of channelrhodopsins (ChRs), which are light-gated cation channels that can be used to depolarize neurons (Nagel et al., 2003, 2005; Boyden et al., 2005; Liewald et al., 2008). Light absorption leads to isomerization of the chromophore all-*trans* retinal (ATR) and opening of the channel pore. A variety of ChRs that are activated by different wavelengths have been discovered or engineered (Guru et al., 2015; Schild and Glauser, 2015; Chang, 2019). This enables multiplexing with both short- or long-wavelength absorbing fluorophores (Wabnig et al., 2015; Hawk et al., 2021; Vierock et al., 2021). In this work, we characterize two different combinations of ChRs with pH-sensitive fluorescent proteins in living *C. elegans* nematodes. We first tested pHuji, a recently described red fluorescent protein, together with the well described blue light-gated ChR2 (Nagel et al., 2005; Shen et al., 2014). To determine whether the increase in fluorescence during stimulation is dependent on SV fusion, we analyzed reduction-of-function mutants of synaptobrevin (SNB-1), which is an essential vesicular soluble N-ethylmaleimide-sensitive-factor attachment receptor (v-SNARE) and thus involved in SV fusion (Nonet et al., 1998; Liu et al., 2018). Similarly, to examine whether the rate of the fluorescence decay after stimulation depended on the rate of SV recycling, we analyzed mutants lacking the established SV recycling factors synaptojanin/UNC-26, endophilin/UNC-57 and synaptotagmin/SNT-1, which are involved in different steps of SV recycling, such as membrane bending and clathrin-uncoating (Jorgensen et al., 1995; Harris et al., 2000; Schuske et al., 2003; Kittelmann et al., 2013; Watanabe et al., 2018; Yu et al., 2018; Mochida, 2022). SNT-1 is the primary sensor of calcium for exocytosis, but also regulates endocytosis, by recruiting adapter complex 2 (AP2) to the plasma membrane (Poskanzer et al., 2003; Yu et al., 2013; Mochida, 2022). The pHuji approach could be used to estimate the extent of SV release but was unable to efficiently report recycling kinetics. We thus swapped both the actuator and the sensor to different excitation wavelengths. We used the recently described red-light activated ChR ChrimsonSA and the well-established green fluorescent pHluorin (Miesenbock et al., 1998; Oda et al., 2018; Seidenthal et al., 2022). Using this combination, we could stimulate and visualize SV exo- and endocytosis in an all-optical, non-invasive manner *in vivo*. We termed this approach the pH-sensitive optogenetic reporter of vesicle recycling (pOpsicle). We tested the pOpsicle method in cholinergic motor neurons and in the glutamatergic/tyraminergic interneuron RIM. pOpsicle should be applicable to most neuronal cell types and is, to our knowledge, the only all-optical approach to study SV recycling using ChRs and pH-sensitive fluorescent proteins in living animals to date. Our approach expands the possibilities to study SV recycling at the *C. elegans* neuromuscular junction (NMJ) which previously could only be done by indirect measurement of postsynaptic effects using electrophysiology, Ca^2+^ imaging, or by (non-)time-resolved EM (Liewald et al., 2008; Kittelmann et al., 2013; Wabnig et al., 2015; Steuer Costa et al., 2017; Yu et al., 2018).

## 2. Materials and methods

### 2.1. Molecular biology

For the expression of SNG-1 fusion constructs and channelrhodopsins in *C. elegans*, the *punc-17* promotor (cholinergic motor neurons) and a short version of the *ptdc-1* promotor (RIM interneurons) were used. **pcDNA3-SypHluorin 4x (S4x)** was a gift from Stephen Heinemann & Yongling Zhu (Addgene plasmid #37005^1^; RRID:Addgene_37005). **pJB14** (*TOPO vector::2xpHluorin*) was generated using the TOPO cloning kit (Thermo Fisher Scientific Inc., USA) by amplifying two copies of pHluorin cDNA from the pcDNA3-SypHluorin 4x (S4x) plasmid with primers oBJ51 (5′-ATATCGAACCGTCTTCAGATATGGATCTAGCCACC-3′) and oBJ62 (5′-TATATTCGCCGTCTTCTCCACCGCATGTGATTCGA GCTCC-3′). **pJB10** (*punc-17::sng-1::unc-54-3*′*UTR*) was generated through Gibson assembly by digesting pRM348 with *BmtI* and *BsiWI* (*punc-17* and backbone), by amplifying pAG52 (*sng-1*) with primers oBJ58 (5′-TCAGGAGGACCCTTGGCTAGATGGAGAACGTGCGTGCTTA TG-3′) and oBJ59 (5′-ATGACTCGAGCTAATAACCATATCCTTC CGACTGAG-3′) and by amplifying pAH03 (*unc-54-3*′*UTR*) with oBJ60 (5′-ATATGGTTATTAGCTCGAGTC ATGGTCGACAAG-3′), and oBJ61 (5′-AAACGCGCGAG ACGAAAGGGCCCAAACAGTTATGTTTGGTATATTGGG-3′). **pJB11** (*punc-17:: sng-1::2xpHluorin::unc-54-3*′*UTR*) was generated by digestion of pJB10 and pJB14 with *BbsI* and subsequent ligation to introduce two copies of pHluorin cDNA into the sequence encoding the first intraluminal loop of SNG-1. **pDisplay-pHuji** was a gift from Robert Campbell (Addgene plasmid #61556^2^; RRID:Addgene_61556). pDisplay-pHuji was amplified using primers oBJ104 (5′-GCAGAAGAAAACCATGGGCTG-3′) and oBJ105 (5′-CAGCCCATGGTTTTCTTCTGC-3′) to remove the *BbsI* restriction site to generate **pJB24**. **pJB25** (*punc-17::sng-1::pHuji::unc-54-3*′*UTR*) was generated *via* Gibson assembly by digesting pJB10 with *BbsI* and amplifying pJB24 with primers oBJ107 (5′-ATATCGAAAAGTCTTCAGGTGGAGGTGGAAGTATGGTGAG CAAGGGCGAG-3′) and oBJ108 (5′-TATATTCGCCGTC TTCGGTGGAGGTGGAAGTCTTGTACAGCTCGTCCATG-3′) which contain the sequence for a GGGGS linker to add in front of the coding region of pHuji. **pJB26** (*punc-17::ChR2(H134R)::myc*) was generated by amplifying *ChR2(H134R)::myc* using primers oBJ113 (5′-GAACGCTAGCACCACTAGATCCATCTAGAG-3′) and oBJ114 (5′-GCATGCTAGCCACCAGACAAGTTGGTAA-3′) which was introduced into pRM348 by restriction digest with *NheI* and subsequent ligation. **pMSE01** (*punc-17::ChrimsonSA::unc-54-3*′*UTR*) was generated by amplifying pDV07 (*punc-17::ChrimsonWT::unc-54-3*′*UTR*) with oMSE16 (5′-CGAGTGGCTGCTGGCTTGCCCCGTGAT-3′) and oMSE017 (5′-ATCACGGGGCAAGCCAGCAGCCACTCG-3′) to introduce the point mutation (S169A). **pMSE23** (*ptdc-1s::ChrimsonSA::unc-54-3*′*UTR*) was generated by amplifying *ptdc-1s* from pXY07 (*ptdc-1s::GFP*) with primers oMSE105 (5′-TCCCGGCCGCCATGGCCGCGATTTCTGTATGAGCCGCCCG-3′) and oMSE106 (5′-AAAGACTTTCGATGAATTACTTGGGCG GTCCTGAAAAATG-3′), amplifying the ChrimsonSA backbone from pMSE01 with oMSE107 (5′-CATTTTTCAGGACCGCCCAAGTAATTCATCGAAAGTCTTT CTATTTTCCGCATCTCTTGTTCAAGGGATTGG-3′) and oMSE108 (5′-CGGGCGGCTCATACAGAAATCGCGGCCATGGC GGCCG-3′) and combined the fragments using Gibson assembly. **pMSE24** (*ptdc-1s::sng-1::pHluorin::unc-54-3*′*UTR*) was generated by amplifying the *sng-1::pHluorin* backbone from pBJ11 with oMSE114 (5′-AGGGTCGACCATGACTCGAGCTAATAACCATATCCTTC-3′) and oMSE115 (5′-GTAATTCATCGAAAGTCTTTCTATTTTCCG CATCTCTTGTTCAAGGGATTGG-3′) and with oMSE108 and oMSE113 (5′-GAAGGATATGGTTATTAGCTCGAGTCATGGT CGACCCT-3′) and fusing these fragments with the *ptdc-1s* using Gibson assembly.

### 2.2. Cultivation of *C. elegans*

*Caenorhabditis elegans* strains were kept under standard conditions on nematode growth medium (NGM) plates seeded with the *Escherichia coli* strain OP50, obtained from the *Caenorhabditis* Genetics Center (CGC, University of Minnesota, USA), at 20°C (Brenner, 1974). The N2 Bristol strain was provided by the CGC and used as wild type. Transgenic animals were generated as described previously (Fire, 1986). An overview of transgenic and mutant strains used or generated in this work can be found in Table 1. For experiments, well-fed L4 larvae were picked ∼18 h before the assays. For RIM experiments, only animals showing marker fluorescence were used. Animals were supplemented with ATR (Sigma-Aldrich, USA) by adding 100 μl OP50 containing 200 μM ATR to 10 ml NGM agar dishes. Experiments were performed on at least two separate days with animals picked from different plates.

**TABLE 1:** *Caenorhabditis elegans* strains.

Strain	Genotype	Source
ZX214	*snt-1(md290)*	CGC (Jorgensen et al., 1995)
ZX307	*snb-1(md247)*	CGC (Miller et al., 1996)
ZX451	*unc-57(e406)*	CGC (Brenner, 1974)
ZX1629	*unc-26(s1710)*	CGC (Harris et al., 2000)
ZX2835	*sng-1(ok234); zxIs138[punc-17::SNG-1::pHuji; punc-17::ChR2(H134R)::myc; pmyo-2::CFP]*	This study
ZX2836	*unc-26(s1710); sng-1(ok234); zxIs138[punc-17::SNG-1::pHuji; punc-17::ChR2(H134R)::myc; pmyo-2::CFP]*	This study
ZX2837	*snt-1(md290); sng-1(ok234); zxIs138[punc-17::SNG-1::pHuji; punc-17::ChR2(H134R)::myc; pmyo-2::CFP]*	This study
ZX2838	*unc-57(e406); sng-1(ok234); zxIs138[punc-17::SNG-1::pHuji; punc-17::ChR2(H134R)::myc; pmyo-2::CFP]*	This study
ZX2850	*snb-1(md247); sng-1(ok234); zxIs138[punc-17::SNG-1::pHuji; punc-17::ChR2(H134R)::myc; pmyo-2::CFP]*	This study
ZX2853	*lite-1(ce314); sng-1(ok234); zxIs138[punc-17::SNG-1::pHuji; punc-17::ChR2(H134R)::myc; pmyo-2::CFP]*	This study
ZX3197	*zxIs152[punc-17::Chrimson(S169A); punc-17::SNG-1::pHluorin; pmyo-2::mCherry]*	This study
ZX3217	*unc-26(s1710); zxIs152[punc-17::Chrimson(S169A); punc-17::SNG-1::pHluorin; pmyo-2::mCherry]*	This study
ZX3218	*snb-1(md247); zxIs152[punc-17::Chrimson(S169A); punc-17::SNG-1::pHluorin; pmyo-2::mCherry]*	This study
ZX3254	*unc-57(e406); zxIs152[punc-17::Chrimson(S169A); punc-17::SNG-1::pHluorin; pmyo-2::mCherry]*	This study
ZX3402	*snt-1(md290); zxIs152[punc-17::Chrimson(S169A); punc-17::SNG-1::pHluorin; pmyo-2::mCherry]*	This study
ZX3422	*zxEx1418[pmyo-2::mCherry; ptdc-1s::ChrimsonSA; ptdc-1s::SNG-1::pHluorin]*	This study

### 2.3. Measurement of *C. elegans* body length

Body length assays were performed as described previously (Liewald et al., 2008; Seidenthal et al., 2022). Briefly, light from a 50 W HBO lamp was filtered with a 450–490 nm bandpass excitation filter for ChR2(H134R) and with a 568.5–591.5 nm filter for ChrimsonSA stimulation. ChR2(H134R) was stimulated at 340 μW/mm^2^. ChrimsonSA was stimulated and tested for crosstalk at a light intensity of 40 μW/mm^2^. Brightfield light was filtered with a 665–715 nm filter to avoid unwanted activation of channelrhodopsins. Videos of single animals were acquired and then analyzed using the WormRuler software (Seidenthal et al., 2022). Body length of each worm was normalized to the 5 s period before stimulation and values >120 or <80% of the initial body length were discarded as these are biomechanically impossible and result from artifacts in the background correction.

### 2.4. Measurement of crawling speed and reversals using the multi-worm-tracker

Videos of crawling animals were acquired as described previously (Vettkötter et al., 2022) and crawling speed measured using the multi-worm-tracker (MWT) setup (Swierczek et al., 2011). Animals were washed three times with M9 buffer to remove OP50 bacteria. They were then transferred to unseeded NGM plates and kept in darkness for 15 min. A light stimulus was applied using a custom-build LED ring (Alustar 3W 30°, ledxon, 623 nm) which was controlled by an Arduino Uno (Arduino, Italy) device running a custom-written Arduino script. Videos were acquired using a high-resolution camera (Falcon 4M30, DALSA) and crawling speed of single animals as well as reversal count (in bins of 10 s) were extracted using “Choreography” software (Swierczek et al., 2011) and summarized using a custom Python script.

### 2.5. Microscopy and imaging

For fluorescence imaging, animals were placed upon 7% agarose pads in M9 buffer. Animals were immobilized using a 20 mM Levamisole-hydrochloride (Sigma-Aldrich, USA) solution in M9 and visualized on an Axio Observer Z1 microscope (Zeiss, Germany) equipped with a 100× oil objective. Fluorescent proteins and channelrhodopsins were excited using a 460 and a 590 nm LED system (Lumen 100, Prior Scientific, UK) coupled *via* a beamsplitter. pHuji and ChR2(H134R) were excited using a double band pass filter (460–500 and 570–600 nm) combined with a 605 nm beam splitter (AHF Analysentechnik, Germany). 460 nm LED light to stimulate ChR2(H134R) was set to 340 μW/mm^2^ intensity. pHuji fluorescence was excited at 280 μW/mm^2^ and filtered using a 615–680 nm emission filter and visualized using an EMCCD camera (Evolve 512 Delta, Teledyne Photometrics, USA). pHluorin and ChrimsonSA were excited using a 450–490/555–590 nm double band pass filter combined with a GFP/mCherry beamsplitter (AHF Analysentechnik, Germany). 590 nm LED light intensity to stimulate ChrimsonSA and excite pHluorin was set to 40 μW/mm^2^. pHluorin fluorescence was filtered using a 502.5–537.5 nm band pass emission filter and visualized using a sCMOS camera (Kinetix 22, Teledyne Photometrics, USA). For increased ChrimsonSA stimulation at 280 μW/mm^2^, we used the same excitation filter as for pHuji experiments but combined with a FITC/TxRed double band pass beamsplitter and a 515–555 nm emission filter (AHF Analysentechnik, Germany). pHluorin fluorescence was again excited with 40 μW/mm^2^. The dorsal nerve cord (DNC) was visualized using the basal pHuji or pHluorin fluorescence. For cholinergic neurons, a region in the posterior third of the animal was imaged, where an abundance of synaptic puncta can be found. For RIM experiments, fluorescent neuronal extensions in the head region were visualized. Videos were captured using the μManager v.1.4.22 software (Edelstein et al., 2014). pHuji – pOpsicle experiments were performed with 50 ms exposure time (20 fps), pHluorin – pOpsicle experiments with 200 ms exposure (5 fps). Stimulation of channelrhodopsins was triggered using a custom written *Autohotkey* script to activate and deactivate LEDs. Representative images displaying entire worms were acquired using a 40× oil objective and stitched together using the ImageJ *Stitching* Plugin (Preibisch et al., 2009). The representative image of RIM neurons (Figure 5A) was made using the *Z Project* function to generate a projection of slices acquired throughout the head region.

### 2.6. Quantification of fluorescence

Example images were processed, and fluorescence was quantified using ImageJ v1.53 (Schindelin et al., 2012). A region of interest (ROI) was placed on the DNC or RIM neuron using the *Segmented Line* tool. Pixel width of the line was adjusted according to the width of the fluorescent signal. A background ROI was set in close proximity to the imaging ROI, inside the worm (but avoiding gut autofluorescence) and fluorescence was quantified using the *Multi Measure* function. XY-drift was corrected using the *Template Matching* ImageJ plugin, if necessary. Animals that moved excessively or drifted in the focal plane were discarded. Fluorescence was normalized to the average fluorescence before stimulation (F_0_) to compare different animals:


$$\frac{△\,F}{F_{0}}=\frac{F-F_{0}}{F_{0}}$$

A custom written python script was used for background subtraction, normalization and (if needed) filtering of animals according to whether they show a strong response during stimulation (available on GitHub^3^). For this, the maximum background corrected fluorescence during stimulation was calculated (as a moving average of 1 s). If this was higher than the average background corrected fluorescence before the stimulation +3 × standard deviations (of background corrected fluorescence before stimulation; minimum of two arbitrary units of fluorescence increase), the animal was counted as a strong responder (adapted from Choi et al., 2021); see Supplementary Figure 6A for example traces that fit or do not fit these parameters; animals not fitting the cut-off showed no discernable light-evoked effect on DNC fluorescence. Also, animals that showed an increase of the fluorescence after the end of the stimulus, or animals showing spontaneous events, were excluded. These measures were necessary for the calculation of fluorescence rise and decay kinetics because data from “non-responders” could not be properly fitted. Fluorescence was not corrected for bleaching since bleaching rates were difficult to estimate robustly in the presence of alternating light protocols. Attempts to correct for bleaching led to a progressive deviation toward the end of the acquisition. However, the measured background fluorescence bleached with a similar rate as the fluorescence in the DNC. Thus, subtraction of background fluorescence was sufficient to correct for bleaching in most cases. Fluorescence signal increases in the pOpsicle assay were calculated using the mean of the normalized fluorescence at the first second after stimulation (±SEM) for pHuji experiments or the mean of seconds 15–20 (±SEM) for pHluorin experiments. Regression analysis, to calculate the rate of fluorescence rise and decay, was performed using Graphpad Prism 9.4.1. The equations “Plateau followed by one-phase exponential association” (1) and “Plateau followed by one-phase exponential decay” (2) were fitted to the fluorescence increase during stimulation and decay after stimulation and the time constants τ calculated for each animal. The curve fits were constrained between minimum and maximum fluorescence values to receive reasonable τ values:


(1) Ift<t:0f(t)=f0;Ift≥t:0f(t)f0+(Max-f0)*(1-e-tτ)

(2) Ift<t:0f(t)f0;Ift≥t:0f(t)(f0-Min)*e-tτ+Min

where. t, time (in seconds); t_0_, timepoint of experimental intervention; set to the first timepoint after the start or stop of stimulation; *f_0_*, value of *f*(*t*) at t_0_; set to 0 (for Equation 1) or maximum fluorescence values (for Equation 2, as a moving average of five frame intervals); Max, maximum fluorescence during or immediately after stimulation as a moving average of five frame intervals (for Equation 1); Min, minimum fluorescence after stimulation as a moving average of 5 s intervals (for Equation 2); τ, time constant (in seconds; higher τ values indicate a slower rise or decay).

As above, each fit was inspected. Individual fits that showed no increase during stimulation were discarded from analysis as they could not be fitted properly (Supplementary Figure 6A). Similarly, data sets that displayed an increase rather than a decay after stimulation were also discarded.

### 2.7. *Caenorhabditis elegans* primary neuronal cell culture

For the preparation of *C. elegans* primary cell culture, established protocols were adapted and modified (Christensen et al., 2002; Strange et al., 2007). Gravid adult worms were grown on enriched peptone plates with nystatin (NEP agar) seeded with Na22 *E. coli* (CGC; Zhang et al., 2011). Worms were washed off the plate using double-distilled water (ddH_2_O) and transferred to 15 ml centrifuge tubes. A total of 2 ml of household bleach as well as 1 ml of 5 M NaOH solution were added to 7 ml of worm suspension. The solution was vortexed for at least 5 min at maximum speed to get rid of adult worm bodies. All the following steps were performed under a sterile workbench. The solution, now containing only eggs, was centrifuged at 500 g for 1 min. Excess liquid was removed, and the pellet was resuspended in ddH_2_O. Washing was repeated three times. The egg pellet was resuspended in 500 μl freshly thawed chitinase (1 U/ml, Sigma-Aldrich, USA) and transferred to a 1.5 ml tube. The tube was placed into a shaker for 90 min at room temperature to digest the chitin shell of the eggs. The chitinase reaction was stopped with 800 μl L-15 full medium (Gibco, USA) containing 10% fetal calf serum (FCS) as well as Pen/Strep (50 U/ml penicillin + 50 μg/ml streptomycin; Sigma-Aldrich, USA). After centrifugation at 900 *g*, excess liquid was discarded, and the pellet was resuspended in 500 μl L-15 full medium. Using a 2 ml syringe with an 18-gauge needle, the solution was aspirated and released back into the tube 15–20 times to dissociate the cells. After dissociation, 1 ml L-15 full medium was added to the tube and taken up into the syringe. With the cell solution inside the syringe, the needle was replaced by a 5 μm filter (Millipore, Germany). The solution was released through the filter into a fresh tube. The original tube was refilled with 1 ml L-15 full medium, the filter was replaced by the needle and the procedure was repeated to release the solution into another tube. This was repeated four to six times, depending on the initial number of eggs (more eggs = less repetitions). Filtered cell solutions were centrifuged at 900 *g* and most of the supernatant was discarded. Cell pellets were resuspended in the remaining medium and pooled. A total of 500–1,000 μl L-15 full medium were added to the suspension and the solution was seeded on 1–2 peanut lectin (Sigma-Aldrich, USA) coated glass bottom petri dishes (ibidi, Germany). Petri dishes were filled with 1 ml L-15 full medium and cells were allowed to adhere for 24 h in a 20°C sterile incubator (Memmert, Germany) before exchanging the medium. Treatment with ATR was performed after two days. Medium was replaced with a 10 μM ATR solution in L15 full medium and the cells incubated for at least 12 h at 20°C. Prior to measurement, cells were washed two times with L-15 full medium without ATR. Neurons were imaged three to four days after seeding. The filter setup was identical to the one used to visualize pHluorin in intact animals, yet neurons were visualized using a 40× objective and 200 ms exposure time. To reduce bleaching during NH_4_Cl treatment frame rate was reduced to one frame every 5 s. The camera shutter and 460 nm LED were controlled using a custom-written Arduino script (kindly provided by Ichiro Aoki). Buffers used to either de-quench or quench pHluorin fluorescence were adapted from Dittman and Kaplan (2006) and added manually by pipetting 1 ml of the respective solution onto the petri dish and then removing the same amount of liquid. Before starting a new acquisition, cells were washed three times using the control saline buffer (pH 7.4). Fluorescence was quantified as in living animals. A ROI was set on top of neurite extensions using the ImageJ “Segmented line” tool with a background ROI set in proximity. The releasable fraction of SVs (RF) was calculated for individual cells as the ratio of the mean normalized fluorescence during optogenetic stimulation to the normalized fluorescence during treatment with NH_4_Cl containing buffer (mean of 85–90 s; Egashira et al., 2016):


$$R\,F=\frac{F_{M\,a\,x\,S\,t\,i\,m}}{F_{N\,H_{4}\,C\,l}}$$

### 2.8. Statistical analysis

Statistical analysis and plotting of graphs were done using Graphpad Prism 9.4.1. Unpaired *t*-tests were performed if two normally distributed datasets were compared and one-way ANOVA (with Dunnett’s or Tukey’s correction) or two-way ANOVA (with Šídák’s correction) for three or more datasets. Fluorescence rise and decay constants were compared using the Mann–Whitney test (for two datasets) and the Kruskal–Wallis tests (with Dunn’s correction) or by applying multiple Mann–Whitney tests (with Holm–Šídák’s correction) for three or more datasets.

## 3. Results

### 3.1. Synaptogyrin-pHuji may be used to quantify SV exocytosis but is unable to efficiently report recycling

To enable an all-optical method for analysis of SV exo- and endocytosis, we used proteins with presumably low optical crosstalk. First, we tested the recently described red fluorescent pHuji protein which shows a 22-fold increase of fluorescence when transferred from intravesicular to extracellular pH (Shen et al., 2014), combined with the blue light-gated ChR2(H134R; hereafter called ChR2) for stimulation of neurotransmission (Nagel et al., 2005). Overlap of the pHuji and ChR2 excitation spectra is minimal (Azimi Hashemi et al., 2014; Shen et al., 2014; Lambert, 2019; Supplementary Figure 1A). *C. elegans* proteins can be targeted to, e.g., cholinergic or GABAergic motor neurons using specific promoters, and for subcellular localization of pHuji we chose the ubiquitous (in neurons) integral SV membrane protein synaptogyrin-1 (SNG-1; Zhao and Nonet, 2001; Abraham et al., 2006). SNG-1 was previously used to target proteins to SVs (Liu et al., 2019; Vettkötter et al., 2022). We inserted pHuji into the second intraluminal loop of SNG-1 (Figure 1) and co-expressed it with ChR2 in cholinergic motor neurons using the promotor of *unc-17*, encoding the vesicular acetylcholine transporter (Alfonso et al., 1993; Miller et al., 1996; Liewald et al., 2008). SNG-1::pHuji could be observed throughout the cholinergic nervous system, i.e., nerve ring, ventral and dorsal nerve cords (VNC/DNC; Supplementary Figure 1B) and dorsal neuronal extensions (Supplementary Figure 1C). Fluorescent puncta in the DNC indicate accumulation of SNG-1::pHuji at NMJs (Sieburth et al., 2005). Next, we analyzed whether neurotransmission can be activated in these animals using ChR2, which typically leads to muscle contraction and reduction of body length through acetylcholine release (Liewald et al., 2008). As expected, blue light caused a decrease of body length of animals when grown in the presence of ATR (Supplementary Figure 1D), but not in no-ATR controls. Next, we quantified fluorescence of SNG-1::pHuji in the posterior DNC where NMJs are abundant (Sieburth et al., 2005) before, during and after ChR2 photostimulation (Supplementary Figures 1C, E). Since pHuji fluorescence was relatively dim, we could not quantify fluorescence during the stimulation period, as blue light increased autofluorescence and/or further excited pHuji, thus erroneously increasing the measured signal. Alternating light protocols also failed, likely due to the photoswitching nature of pHuji (Liu et al., 2021). However, we could compare the fluorescence before and after stimulation. Animals treated with ATR showed a significantly increased fluorescence after stimulation (19 ± 3% ΔF/F_0_), compared to animals without ATR (2 ± 2%, ^***^p < 0.001; Supplementary Figure 1E). This increase gradually declined toward the fluorescence level in animals grown without ATR, most likely indicating recycling of externalized SNG-1::pHuji (and thus of SVs). We termed the pHuji-ChR2 combination “red pOpsicle.” To determine whether the red-pOpsicle assay reports on altered SV exo- and endocytosis, we crossed the line carrying the ChR2 and SNG-1::pHuji transgene into mutants known to affect the SV cycle (see section “1. Introduction”; Supplementary Figure 2). Compared to wild type, *snb-1(md247)* mutants showed a significantly lower fluorescence increase after stimulation with blue light [wild type: 15 ± 2%, *snb-1(md247)*: 2 ± 2%; Supplementary Figures 2A, B], indicating lower amounts of SVs containing SNG-1::pHuji being exocytosed. Deletion mutants of SV recycling factors, *unc-26(s1710)* and *unc-57(e406)*, displayed a higher increase in pHuji fluorescence than wild type animals [Supplementary Figure 2C: wild type 17 ± 3%, *unc-26(s1710)* 31 ± 5%, *p < 0.05; Supplementary Figure 2D: wild type 16 ± 3%, *unc-57(e406)* 30 ± 4%, *p < 0.05]. This was surprising since both mutants were previously shown to be depleted of ready-to-release SVs and should therefore not be able to externalize as many SVs as wild type animals (Harris et al., 2000; Schuske et al., 2003). Nevertheless, they showed prolonged increased fluorescence during acquisition compared to wild type. *snt-1(md290)* knockout mutants displayed a similar signal increase and decay trend as wild type [Supplementary Figure 2E: wild type 14 ± 3%, *snt-1(md290)* 18 ± 3%, ns *p* > 0.05]. We calculated the kinetics of fluorescence decay after stimulation using a one-phase exponential decay model (Supplementary Figures 2F–H). However, no significant difference in the rate of the fluorescence decay was observed. We thus wondered if the pHuji/ChR2 combination works properly. One concern was that photophobic reactions to blue light, mediated by the photoreceptor LITE-1, may affect the measured fluorescence increase and recycling rate (Edwards et al., 2008). However, we did not observe any significant difference between *lite-1(ce314)* knockout mutants and wild type animals in the pOpsicle assay, independent of ATR treatment (Supplementary Figures 2I, J). We suggest that the previously reported photo-switching behavior of pHuji in blue light (Liu et al., 2021) likely explains the slight increase in fluorescence in animals without ATR after stimulation and could also influence the decay kinetics in animals with ATR (Supplementary Figures 1E, 2I). Since pHuji appeared unable to properly track SV recycling, we investigated another protein combination for the pOpsicle assay.

### 3.2. Improving pOpsicle through combination of pHluorin with ChrimsonSA

**FIGURE 2:**
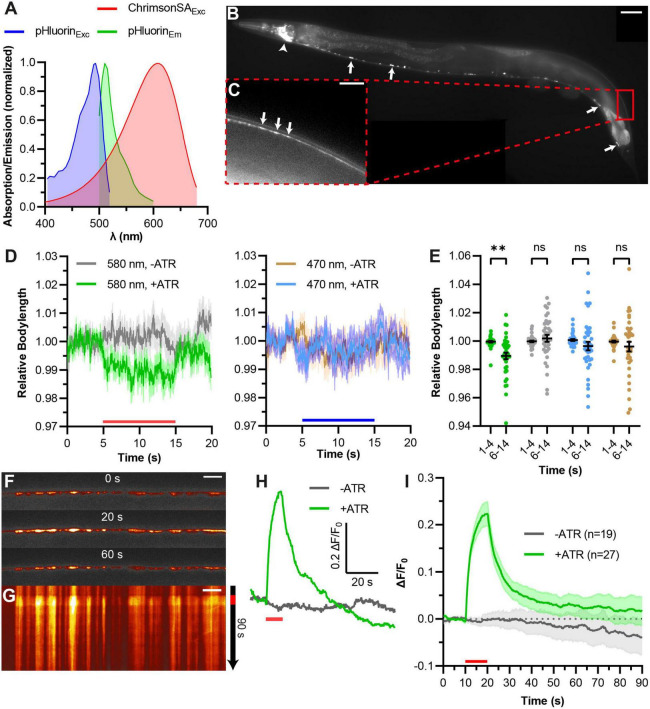
Combining ChrimsonSA with pHluorin (“green pOpsicle”) for stimulation and visualization of exo- and endocytosis. **(A)** Relative excitation and emission spectra of ChrimsonSA and super-ecliptic pHluorin, normalized to the maximum absorption/emission amplitude. **(B)** Representative image of *C. elegans* expressing SNG-1::pHluorin in cholinergic neurons. Arrows represent cell bodies of A- and B-type motor neurons (ventral nerve cord). Arrowhead represents cholinergic neurons in the head ganglia. 40× magnification. Scale bar, 50 μm. **(C)** Enlarged view of the DNC in panel **(B)**. Arrows represent fluorescent puncta, representing *en passant* synaptic terminals. Scale bar, 10 μm. **(D)** Mean relative body length (±SEM) of animals expressing ChrimsonSA and pHluorin optionally treated with ATR, as indicated, normalized to the average body length before stimulation. A 10 s continuous light pulse (580 or 470 nm as indicated, 40 μW/mm^2^; indicated by red and blue bars) was applied after 5 s. Number of animals (*n*), accumulated from *N* = 3 biological replicates. **(E)** Statistical analysis of animals shown in panel **(D)**. Each dot represents the mean normalized body length of a single animal during the indicated period. Two-way ANOVA with Šídák’s correction (^ns^*p* > 0.05, ^**^p < 0.01). **(F)** Representative images of pHluorin fluorescence in the DNC of an animal treated with ATR at different time points during the pOpsicle assay, as indicated. A 10 s continuous light pulse (590 nm, 40 μW/mm^2^) was applied after 10 s. The ImageJ *Smart* Look-Up-Table was used. 100× magnification. Scale bar, 5 μm. **(G)** Kymograph representing the change in fluorescence of the DNC represented in panel **(F)** over a time course of 90 s. The red bar indicates the period of light stimulus. Scale bar, 5 μm. **(H)** Representative traces of normalized DNC fluorescence of individual animals with or without ATR. A 10 s continuous light pulse (590 nm, 40 μW/mm^2^) was applied after 10 s (red bar). **(I)** Mean (±SEM) change in DNC fluorescence of animals supplemented with and without ATR. A 10 s continuous light pulse (590 nm, 40 μW/mm^2^) was applied after 10 s (red bar). Number of animals (*n*), accumulated from *N* = 4 (+ATR), and *N* = 3 (–ATR) biological replicates.

We tested the more commonly used green fluorescent pHluorin as a pH-sensitive fluorophore which moreover features brighter fluorescence (Miesenbock et al., 1998; Li et al., 2022). Since pHluorin has overlapping excitation spectra with ChR2, we needed to exchange ChR2 with a red light activated channelrhodopsin. The recently described ChrimsonSA (for super red-shifted and accelerated), which is a mutated variant (S169A) of Chrimson, seemed to be a suitable candidate (Oda et al., 2018). Previously, we could show that ChrimsonSA can be used to depolarize *C. elegans* motor neurons upon stimulation with red light (Seidenthal et al., 2022; Figure 2A). Two copies of pHluorin were inserted into the second intraluminal loop of SNG-1 as multiple pHluorin insertions have been shown to increase the signal-to-noise ratio (Zhu et al., 2009). We co-expressed this fusion construct with ChrimsonSA in cholinergic motor neurons and observed basal pHluorin fluorescence throughout the cholinergic nervous system (Figure 2B). SNG-1::pHluorin also localizes to neuronal extensions of DA and DB neurons innervating dorsal muscle cells (Figure 2C). Fluorescent puncta in the DNC indicate an accumulation of pHluorin at NMJs. We then tested for crosstalk of pHluorin imaging light with ChrimsonSA activation using contraction assays (Figures 2D, E). The intensity of blue light used to visualize pHluorin (470 nm, 40 μW/mm^2^) did not lead to a significant decrease in body length of animals treated with ATR. In contrast to this, 40 μW/mm^2^ of 580 nm light was sufficient to trigger contraction of body wall muscles. This indicates that ChrimsonSA is functioning properly to depolarize cholinergic motor neurons yet is not pre-activated by blue light. Next, we performed pOpsicle assays with pHluorin animals (“green pOpsicle”). We observed spontaneous pHluorin increases in some animals, independent of treatment with ATR or light stimulation (Supplementary Figure 3A), verifying that pHluorin imaging can report on exocytosis. During photostimulation, DNC fluorescence gradually increased by 21 ± 2% upon continuous 590 nm stimulation in ATR-treated animals (Figures 2F–I, see Supplementary Figure 3B for a representation of all experiments, and Supplementary Movie 1). The signal approached a plateau after ∼10 s, indicating that most SNG-1::pHluorin was externalized at this point. Animals without ATR showed no increase upon stimulation (0 ± 1%, ^***^p < 0.001; Figures 2H, I). The increase in fluorescence was especially high in synaptic puncta along the DNC, indicating locations of highly active SV release sites (Figures 2F, G). Following the end of the stimulation, like in red pOpsicle experiments, the signal gradually decreased toward levels before stimulation. We also tested a higher light intensity to stimulate ChrimsonSA (280 μW/mm^2^) and observed a slightly higher increase in fluorescence yet overall similar trend (Supplementary Figure 3C; 30 ± 3%, *p < 0.05 compared to 40 μW/mm^2^). Similarly, stronger light stimulation led to a linearly increasing contraction in behavioral experiments (Supplementary Figure 3D). For subsequent analyses we used 40 μW/mm^2^ since it seemed sufficient to release most SVs and stronger stimulation may trigger unphysiological effects.

To further characterize parameters of the “green pOpsicle” assay, e.g., releasable fraction of total pHluorin, we replicated it in neurons of primary cell cultures from dissociated *C. elegans* embryos (Supplementary Figures 4A, B). pHluorin fluorescence could be observed in neurite extensions and in cell bodies, surrounding nuclei (Supplementary Figure 4A). This fluorescence increased in some neurons when illuminated (and when treated with ATR) even though the signal size was lower than in intact animals, where this was the case for each individual recording (Figure 2I and Supplementary Figure 4B). A total of 18 of 52 neurons showed a strong response (for a definition of “strong response,” see section “Materials and methods”). Next, we calculated the fraction of releasable SVs by ChrimsonSA stimulation. To this end, we first stimulated cultured neurons with light and then applied a pH 7.4 NH_4_Cl buffer (Supplementary Figure 4C), which can be used to increase the pH within SVs and calculate the SV-associated portion of pHluorins (Sankaranarayanan et al., 2000; Dittman and Kaplan, 2006; Rose et al., 2013; Egashira et al., 2016). To minimize bleaching, we reduced the exposure to blue light by decreasing the frame rate of pHluorin acquisition. When exposed to NH_4_Cl (pH 7.4), pHluorin fluorescence in neurites rapidly increased (Supplementary Figures 4C–E). The same culture was then treated with a low pH buffer (pH 5.6) to quench the surface fraction of pHluorin (Supplementary Figures 4C, F). The fluorescence immediately decreased to levels below the basal fluorescence, demonstrating the pH-dependency of pHluorin fluorescence. The fraction of releasable SVs was determined by calculation of the ratio of maximum fluorescence during light stimulation and during NH_4_Cl treatment (Supplementary Figure 4G): 10 s continuous light stimulation released 20.20% (±3.72%) of SNG-1::pHluorin molecules attached to SVs, which is comparable to previous measurements in hippocampal slices that used synaptophysin::pHluorin as a reporter (Rose et al., 2013). To test a different, less vigorous stimulation regime, which may be more reminiscent to endogenous activity, we applied 2 Hz pulsed light (Supplementary Figure 4C). This led to a reduced release of SVs compared to continuous light (Supplementary Figure 4G, 10.61 ± 1.87%). Even though the fraction of releasable SVs by optogenetic stimulation is within a typical range compared to other reports, we conclude that in intact animals the signal size is higher and the assay more reliable in living animals indicating that a higher portion of SVs is exocytosed (Figure 2I and Supplementary Figure 4B).

### 3.3. Quantification of SV exo- and endocytosis kinetics by the pHluorin pOpsicle assay

**FIGURE 3:**
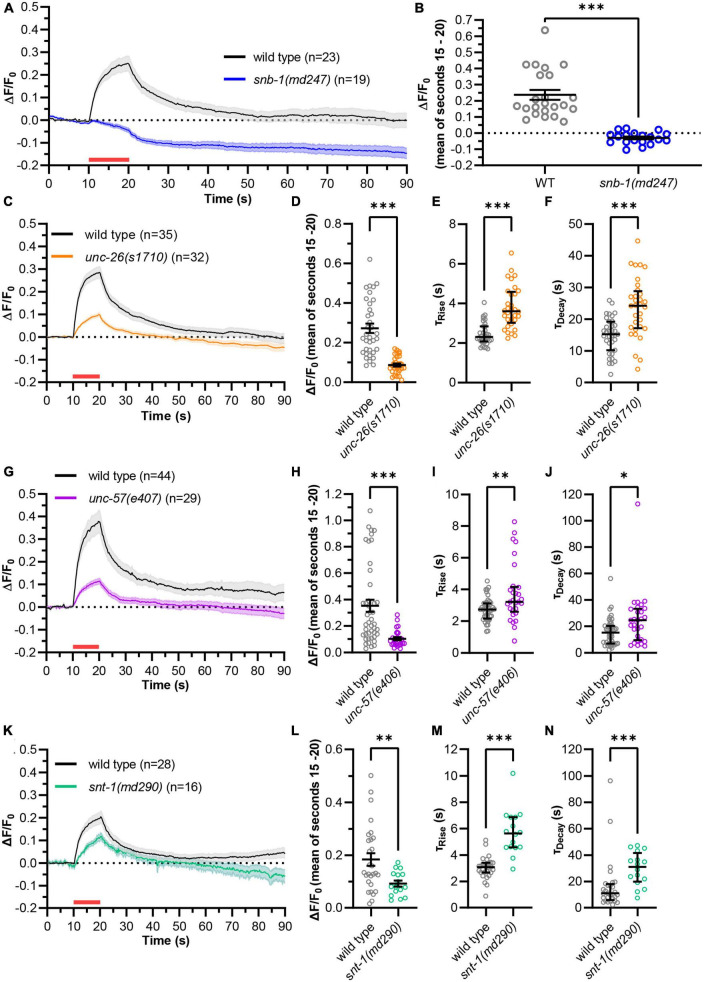
“Green” pOpsicle reports on mutations affecting SV fusion and endocytosis. **(A,C,G,K)** Mean (±SEM) change of fluorescence of SNG-1::pHluorin co-expressed with ChrimsonSA in cholinergic motor neurons. DNC of wild type and mutant animals, as indicated. A 10 s continuous light pulse (590 nm, 40 μW/mm^2^; indicated by a red bar) was applied after 10 s. Number of animals (*n*), accumulated from *N* = 4–6 biological replicates. **(B,D,H,L)** Fluorescent signal of individual wild type and mutant animals at the end of stimulation (15–20 s). Mean (±SEM). Unpaired *t*-test (^**^p < 0.01, ^***^p < 0.001). **(E,I,M)** Calculated fluorescence rise constants of single animals using a one-phase exponential fit during stimulation (10–20 s). Median with interquartile range. Mann–Whitney test (^**^p < 0.01, ^***^p < 0.001). **(F,J,N)** Calculated fluorescence decay constants of single animals using a one-phase exponential fit after stimulation (20–90 s). Median with interquartile range. Mann–Whitney test (*p < 0.05, ^***^p < 0.001). In panels **(C–N)**, animals showing an increase <3 standard deviations during stimulation, or no decay of fluorescence following stimulation, were excluded [wild type: 4 of 111 animals, *unc-26(s1710)*: 7 of 39, *unc-57(e406)*: 18 of 47, *snt-1(md290)*: 23 of 39].

Next, we used green pOpsicle to analyze mutants affecting SV fusion (*snb-1(md247), snt-1(md290)*) and/or recycling (*unc-26(s1710)*, *unc-57(e406), snt-1(md290)*) (Figure 3). In *snb-1(md247)* mutants, the signal increase during stimulation was almost completely abolished (Figures 3A, B and Supplementary Figure 5A). Only 2 of 19 animals showed a relevant response (compared to 23/23 in wild type), demonstrating that the increase of fluorescence during stimulation reports on SV exocytosis. Synaptojanin (*unc-26(s1710)*) mutant animals also exhibited a reduced signal compared to wild type (Figures 3C, D and Supplementary Figure 5B). Thus, green pOpsicle faithfully revealed the expected depletion of SVs in *unc-26(s1710)* mutants (Harris et al., 2000), unlike pHuji that displayed a higher fluorescence after stimulation (Supplementary Figure 2C). To allow calculating the kinetics of fluorescence rise and decay, animals which showed no significant increase in fluorescence or no decay after stimulation were removed from our analyses (Supplementary Figure 6A). We observed a significantly slower rise of fluorescence in *unc-26(s1710)* mutant compared to wild type animals, as could be seen by the increased time constants of the signal curves obtained from single animals, when fitted to a one-phase exponential association kinetic (τ_Rise, wild type_ = 2.3 s, τ_Rise,_ *_*unc–26*_* = 3.6 s; Figure 3E). This could indicate an exocytosis defect, or it could be due to the reduced number of SVs that are available for fusion in the *unc-26(s1710)* mutant (Dong et al., 2015). The kinetics of the fluorescence decay after stimulation showed a strong reduction, as expected for the SV recycling mutant (Figure 3F). Consequently, the calculated time constants of one-phase exponential decay were significantly increased in *unc-26(s1710)* mutants (τ_Decay, wild type_ = 15.2 s, τ_Decay,_ *_*unc–26*_* = 24.2 s), closely matching previous results measured in mammalian cortical neurons lacking synaptojanin after strong stimulation (Cao et al., 2017). Wild type animals on the other hand showed decay time constants which are in the range of previous measurements in *C. elegans* sensory neurons or mammalian hippocampal neurons (Kwon and Chapman, 2011; Ventimiglia and Bargmann, 2017; Watanabe et al., 2018; Li et al., 2022). Endophilin (*unc-57(e406)*) mutants exhibited similar trends as *unc-26(s1710)* with a significantly reduced signal and significantly slower association and decay kinetics compared to wild type (τ_Rise, wild type_ = 2.7 s, τ_Rise,_ *_*unc–57*_* = 3.3 s, τ_Decay, wild type_ = 15.2 s, τ_Decay,_ *_*unc–57*_* = 24.7 s; Figures 3G–J and Supplementary Figure 5C). The time constants obtained for *unc-57(e406)* mutants closely matched those of *unc-26(s1710)* mutants, in line with the function of synaptojanin and endophilin in similar cellular processes (Harris et al., 2000; Dong et al., 2015; Watanabe et al., 2018). Synaptotagmin [*snt-1(md290)*] mutants also displayed a smaller increase in fluorescence (Figures 3K, L and Supplementary Figure 5D). The time constants of fluorescence rise were significantly increased in mutant animals (τ_Rise,wild type_ = 3.1 s, τ_Rise,snt–1_ = 5.6 s; Figure 3M), demonstrating the role of SNT-1 in SV fusion (Liewald et al., 2008; Yu et al., 2013). We further observed a significantly delayed decrease of fluorescence after stimulation, indicating that SNT-1 is involved in SV recycling at *C. elegans* NMJs (Poskanzer et al., 2003; Mochida, 2022; τ_Decay, wild type_ = 11.1 s, τ_Decay,_ *_*snt–1*_* = 31.1 s; Figure 3N). Since the increase in fluorescence was generally lower in mutant animals, we wondered whether higher time constants of decay were caused by a lower activation of synaptic transmission rather than decreased SV recycling rates. We thus compared decay time constants of individual animals with the respective signal size. However, there was no significant correlation within any of the analyzed genotypes (Supplementary Figures 6B–D). Therefore, recycling rates likely do not depend on the amount of SV fusion in this assay.

### 3.4. Pulsed stimulation to potentially access different recycling mechanisms

**FIGURE 4:**
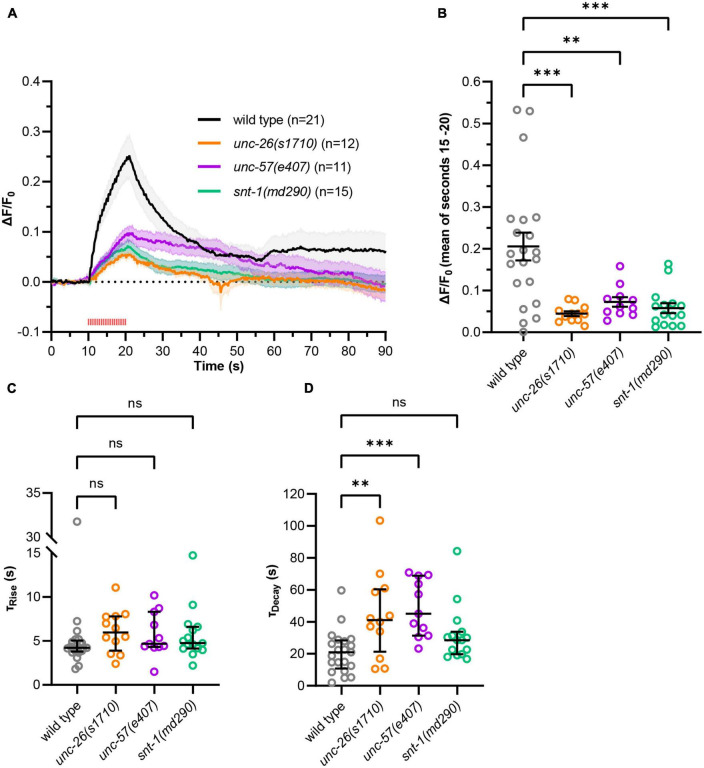
Using pulsed, more physiological optogenetic stimulation. **(A)** As in Figure 3, but using 2 Hz pulsed light stimulation (100 ms pulses, 590 nm, 40 μW/mm^2^, red tick marks) applied after 10 s for 10 s. Using this stimulation regime, more animals were excluded from analysis [wild type: 17 of 38 animals, *unc-26(s1710)*: 18 of 30, *unc*-57*(e406)*: 22 of 33, *snt-1(md290)*: 39 of 54] accumulated from *N* = 5–6 biological replicates. **(B)** Fluorescent signal of individual wild type and mutant animals at the end of stimulation (15–20 s). Mean (±SEM). One-way ANOVAs with Dunnett’s correction (^**^p < 0.01, ^***^p < 0.001). **(C)** Calculated fluorescence rise constants of single animals using a one-phase exponential fit during stimulation (10–20 s). Median with interquartile range. Kruskal–Wallis test with Dunn’s correction (^ns^*p* > 0.05). **(D)** Calculated fluorescence decay constants of single animals using a one-phase exponential fit after stimulation (20–90 s). Median with interquartile range. Kruskal–Wallis test with Dunn’s correction (^ns^*p* > 0.05, ^**^p < 0.01, ^***^p < 0.001).

Continuous photostimulation induces maximal depolarization and transmitter release, likely causing bulk endocytosis as the extreme form of ultrafast endocytosis (Kittelmann et al., 2013). To assess whether less vigorous, possibly more physiological activation also affects slower recycling, we applied 2 Hz pulsed stimulation (100 ms light pulses; Figure 4). The mutant strains again showed significantly reduced signal amplitudes compared to wild type (Figures 4A, B and Supplementary Figure 7) but no significantly increased time constants of fluorescence rise (τ_Rise, wild type_ = 4.2 s, τ_Rise,_ *_*unc–26*_* = 6.0 s, τ_Rise,_ *_*unc–57*_* = 4.7 s, τ_Rise,_ *_*snt–1*_* = 4.7 s; Figure 4C). When comparing pulsed and continuous stimulation we observed a tendency toward decreased fluorescence amplitudes and significantly increased rise time constants, indicating that pulsed stimulation leads to a reduced activation of neurotransmission (Supplementary Figures 8A, B). For the recycling phase, pulsed stimulation again resulted in significantly larger decay time constants in *unc-57(e406)* and *unc-26(s1710)* mutants compared to wild type (τ_*Decay,wild type*_ = 20.9 s, τ_*Decay*,_ *_*unc–26*_* = 41.1 s, τ_*Decay*,_ *_*unc–57*_* = 45.1 s; Figure 4D), however, *snt-1(md290)* mutant animals showed no significant difference (τ_*Decay*,_ *_*snt–1*_* = 28.6 s). Possibly, SNT-1 is dispensable for recycling at lower levels of stimulation. Decay time constants for pulsed stimulation were significantly increased in *unc-57(e406)* and *unc-26(s1710)* mutants indicating that a compensatory mechanism, which is independent of endophilin and synaptojanin, may be activated after strong stimulation (Supplementary Figure 8C).

**FIGURE 5:**
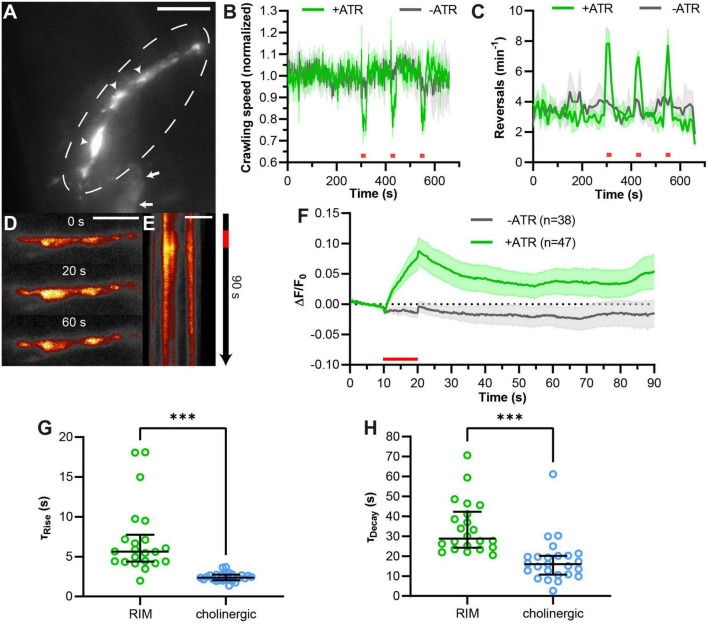
pOpsicle assay in the RIM interneuron pair. **(A)** Representative *Z*-projected image of *C. elegans* expressing SNG-1::pHluorin in RIM neurons using the promotor of *tdc-1*. 40× magnification. Arrows represent cell bodies of RIM neurons. Circle represents RIM axon in the nerve ring. Arrowheads represent fluorescent puncta, representing *en passant* synaptic terminals. 40× magnification. Scale bar, 5 μm. **(B)** Mean crawling speed (±SEM) of animals expressing ChrimsonSA and pHluorin in RIM neurons normalized to the average before the first light pulse. Three 20 s light pulses (623 nm, 400 μW/mm^2^) were applied at 300, 420, and 540 s as indicated by red bars. **(C)** Mean (±SEM) number of reversals per minute per animal. Light stimulation as in panel **(B)**. In panels **(B,C)**, *N* = 3 populations of animals were tested. **(D)** Representative images acquired at different time points during the pOpsicle assay, pHluorin fluorescence in RIM neurons, animal treated with ATR. A 10 s continuous light pulse (590 nm, 40 μW/mm^2^) was applied after 10 s. The ImageJ *Smart* Look-Up-Table was used. 100× magnification. Scale bar, 5 μm. **(E)** Kymograph representing the change in fluorescence in RIM neurons as shown in panel **(D)** over a time course of 90 s. The red bar indicates the period of light stimulus. Scale bar, 5 μm. **(F)** Mean (±SEM) pHluorin fluorescence in RIM neurons of animals with and without ATR. A 10 s continuous light pulse (590 nm, 40 μW/mm^2^) was applied after 10 s. Number of animals (*n*), accumulated from *N* = 5 (+ATR) or *N* = 4 (–ATR) biological replicates. **(G)** Comparison of fluorescence rise constants of single animals expressing pHluorin and ChrimsonSA in RIM neurons or in cholinergic neurons, using a one-phase exponential fit during stimulation (10–20 s). Median with interquartile range. Mann–Whitney test (^***^p < 0.001). **(H)** Comparison of fluorescence decay constants of single animals as in panel **(G)**, using a one-phase exponential fit after stimulation (20–90 s). Median with interquartile range. Mann–Whitney test (^***^p < 0.001). **(G,H)** Only animals showing a strong response during stimulation and a decay of fluorescence after stimulation were taken into consideration (RIM: 22 of 47 animals, cholinergic neurons: 25 of 27 animals as depicted in Figure 2).

### 3.5. pOpsicle reports on SV turnover in the single pair of RIM interneurons

While the green pOpsicle assay worked well in cholinergic motor neurons, it remained to be shown that this system works in other neuronal cell types. The RIM interneuron pair integrates signals from sensory neurons to regulate forward and reversal locomotion, using gap junctions as well as glutamate and tyramine signaling (Piggott et al., 2011; Li et al., 2020; Sordillo and Bargmann, 2021). We expressed SNG-1::pHluorin and ChrimsonSA in RIM using the *tdc-1* promotor. Green fluorescence could be observed in neuronal extensions surrounding the pharynx, suggesting correct localization of SNG-1::pHluorin (Figure 5A). To explore ChrimsonSA functionality in this neuron pair, we measured animal crawling speed (Figure 5B). Optogenetic depolarization of RIM neurons previously induced reversals and reduced crawling speed (Guo et al., 2009; Li et al., 2020; Sordillo and Bargmann, 2021). Consistent with this, illumination with red light slowed down crawling speed of RIM pOpsicle animals treated with ATR and the number of reversals was increased (Figures 5B, C). This indicated that ChrimsonSA depolarized RIM neurons when activated. Consequently, SNG-1::pHluorin fluorescence in synaptic puncta was significantly increased by ChrimsonSA activation (Figures 5D, E, Supplementary Figure 9, and Supplementary Movie 2), while control animals without ATR showed no change (+ATR 6 ± 2%, −ATR −1 ± 1%, ^***^p < 0.001; Figure 5F). The signal increase was significantly slower than in cholinergic neurons (τ_Rise, RIM_ = 5.7 s, τ_Rise, cholinergic_ = 2.4 s; Figure 5G), as was the decay of fluorescence following the end of stimulation (τ_Decay, RIM_ = 42.3 s, τ_Decay, cholinergic_ = 16.0 s; Figure 5H). These results indicate that different classes of neurons may have diverging kinetics of SV exo- and endocytosis in *C. elegans*. Thus, the pOpsicle assay can be adapted to different neuronal cell types.

## 4. Discussion

Here, we present the first all-optical method to investigate SV recycling *in vivo* by combining pH-sensitive fluorescent proteins with ChRs. With pOpsicle, factors that influence the extent and rate of exo- and endocytosis can be investigated with minor experimental effort and equipment. We described two approaches using different pH-sensitive fluorescent proteins. pHuji could only be used to quantify the extent of exocytosis after stimulation. The low quantum yield and photoswitching behavior of this protein influenced the emitted fluorescence in a way that precluded quantification of exo- and endocytosis kinetics (Shen et al., 2014; Liu et al., 2021). Using pHluorin and ChrimsonSA, however, solved these problems, enabling calculation of fluorescence rise and decay time constants, which characterize different rates of exo- and endocytosis (Li et al., 2022). Neuronal primary culture of pHluorin expressing neurons could further open the way for investigation of exocytosis independent of SV recycling, e.g., by applying pharmacological agents such as bafilomycin A to inhibit SV acidification (Subramanian and Morozov, 2011; Li et al., 2022).

The pOpsicle system should be applicable to various neuronal cell types with minor modifications as exemplified by expression in cholinergic neurons and RIM interneurons. Establishing pOpsicle in various cell types could unveil disparities in SV exo- and endocytosis between different neuron classes, as has been shown for sensory neurons in *C. elegans* (Ventimiglia and Bargmann, 2017). Hitherto, electrophysiological recordings or Ca^2+^ imaging in body wall muscles were the method of choice to quantify neurotransmitter release in *C. elegans* in a time-resolved manner (Liewald et al., 2008; Wabnig et al., 2015). However, these only report postsynaptic effects which might be altered by unrelated phenomena such as neurotransmitter-receptor upregulation (Hammond-Weinberger et al., 2020). By using pOpsicle, a direct observation of the presynaptic SV cycle was achieved. This way, we could observe slowed SV fusion and endocytosis in RIM interneurons compared to cholinergic motor neurons. The faster release and recycling in cholinergic neurons may be in line with their function in mediating locomotion, and the likely high SV turnover needed, while the slower release rate could be important to the dual role of RIM in regulating reversal behavior (Li et al., 2020; Sordillo and Bargmann, 2021; Bach et al., 2023). While RIM promotes reversals through activation AVA and AVE neurons *via* gap junctions, and by mutual interaction with RIS, glutamate signaling inhibits reversal probability by reducing the amplitude of Ca^2+^ spikes within AVA and AVE and tyramine inhibits RIS (Li et al., 2020; Bach et al., 2023). A delayed release of glutamate and tyramine from RIM may thus promote a fast reaction to noxious stimuli by initiation of reversals. Observation of pHluorin dynamics in freely moving worms may solve this issue. However, we note that it was imperative for the assay to work that the animals were kept as immobile as possible.

With green pOpsicle, we could reveal differences in recycling kinetics of synaptojanin and endophilin knockout mutants between continuous and pulsed stimulation. Discrepancies in the rate of recycling may be caused by different degrees of stimulation which trigger distinct routes of SV retrieval (Watanabe and Boucrot, 2017). Activity-dependent bulk endocytosis (ADBE) after strong optogenetic stimulation was shown to occur in *unc-57(e406)* and *unc-26(s1710)* knockout mutants and may be the main pathway of retrieval after continuous stimulation (Kittelmann et al., 2013; Nicholson-Fish et al., 2016; Yu et al., 2018). Moderate, pulsed stimulation however may trigger other endocytic mechanisms such as ultrafast and clathrin-mediated endocytosis, which are dependent on endophilin and synaptojanin (Milosevic et al., 2011; Watanabe et al., 2018). *snt-1(md290)* mutants displayed more severe recycling defects after strong stimulation, indicating that it is dispensable for recycling at lower activity. This is in stark contrast to previous results in mammalian hippocampal neurons in which synaptotagmin promotes slow small-scale endocytosis, while inhibiting bulk retrieval during sustained neurotransmission (Chen et al., 2022). Although a role of SNT-1 in the slow clathrin-mediated endocytosis is likely also in *C. elegans* and simply not efficiently reported by pOpsicle, a role in inhibition of bulk endocytosis might not be conserved between nematodes and mammals. Previously, *snt-1(md290)* mutants have shown earlier fatigue of postsynaptic currents during strong optogenetic depolarization of cholinergic motor neurons indicating a compensatory SV recycling defect during strong sustained neurotransmission (Liewald et al., 2008; Wabnig et al., 2015).

Finally, SNG-1::pHluorin can further be used as a sensor for spontaneous neuronal activity and SV endocytosis in *C. elegans*, independent of optogenetic stimulation (Supplementary Figure 3A). This opens the way for multiplexing with other fluorescent reporters of neuronal activity such as genetically encoded Ca^2+^ or voltage indicators (Dreosti and Lagnado, 2011; Jackson and Burrone, 2016; Azimi Hashemi et al., 2019). The combination of simultaneous imaging of SV dynamics and membrane potential changes could help to unravel the complicated interplay of interneurons in the control of locomotion by differentiating between electrical and chemical transmission.

## Data availability statement

The original contributions presented in this study are included in the article/Supplementary material, further inquiries can be directed to the corresponding author.

## Author contributions

MS, BJ, and XZ created the plasmids and generated the strains. MS performed the pOpsicle, cell culture, and contraction assays. MW tested the ChrimsonSA in cholinergic neurons. NS and NE generated and tested the ChrimsonSA/pHluorin strains. NR generated the primary neuronal cell cultures. MS, BJ, and AG designed and coordinated the study. MS and AG wrote the manuscript. AG supervised the work. All authors read and approved the final manuscript.

## Footnotes

1
http://n2t.net/addgene:37005

2
http://n2t.net/addgene:61556

3
https://github.com/MariusSeidenthal/pHluorin_Imaging_Analysis
