# Supplementary material for: pOpsicle: An all-optical reporter system for synaptic vesicle recycling combining pH-sensitive fluorescent proteins with optogenetic manipulation of neuronal activity
Source: Front Cell Neurosci. 2023 Mar 31;17:1120651. doi: 10.3389/fncel.2023.1120651 (PMC10102542; doi:10.3389/fncel.2023.1120651)
Supplement: Supplementary file 1 [file Data_Sheet_1.PDF]

Supplementary Material

pOpsicle: An all-optical reporter system for synaptic vesicle recycling combining pH-sensitive fluorescent proteins with optogenetic manipulation of neuronal activity

Marius Seidenthal[1,2], Barbara Jánosi[1,2], Nils Rosenkranz[1,2], Noah Schuh[1,2], Nora Elvers[1,2], Miles Willoughby[1,2], Xinda Zhao[1,2], Alexander Gottschalk[*,1,2]

*** Correspondence:**
Alexander Gottschalk
a.gottschalk@em.uni-frankfurt.de

1 Supplementary Data

Supplementary Movie 1. Representative video of the DNC in an animal expressing SNG-1::pHluorin and ChrimsonSA in cholinergic neurons treated with ATR. A 10 s continuous light pulse (590 nm, 40 µW/mm2) was applied after 10 s as represented by red dot. The ImageJ Smart Look-Up-Table was used. 100 x magnification. Scale bar, 5 µm.

Supplementary Movie 2. Representative video of RIM neurons in an animal expressing SNG-1::pHluorin and ChrimsonSA in cholinergic neurons treated with ATR. A 10 s continuous light pulse (590 nm, 40 µW/mm2) was applied after 10 s as represented by red dot. The ImageJ Smart Look-Up-Table was used. 100 x magnification. Scale bar, 5 µm.

2 Supplementary Figures and Tables

2.1 Supplementary Figures

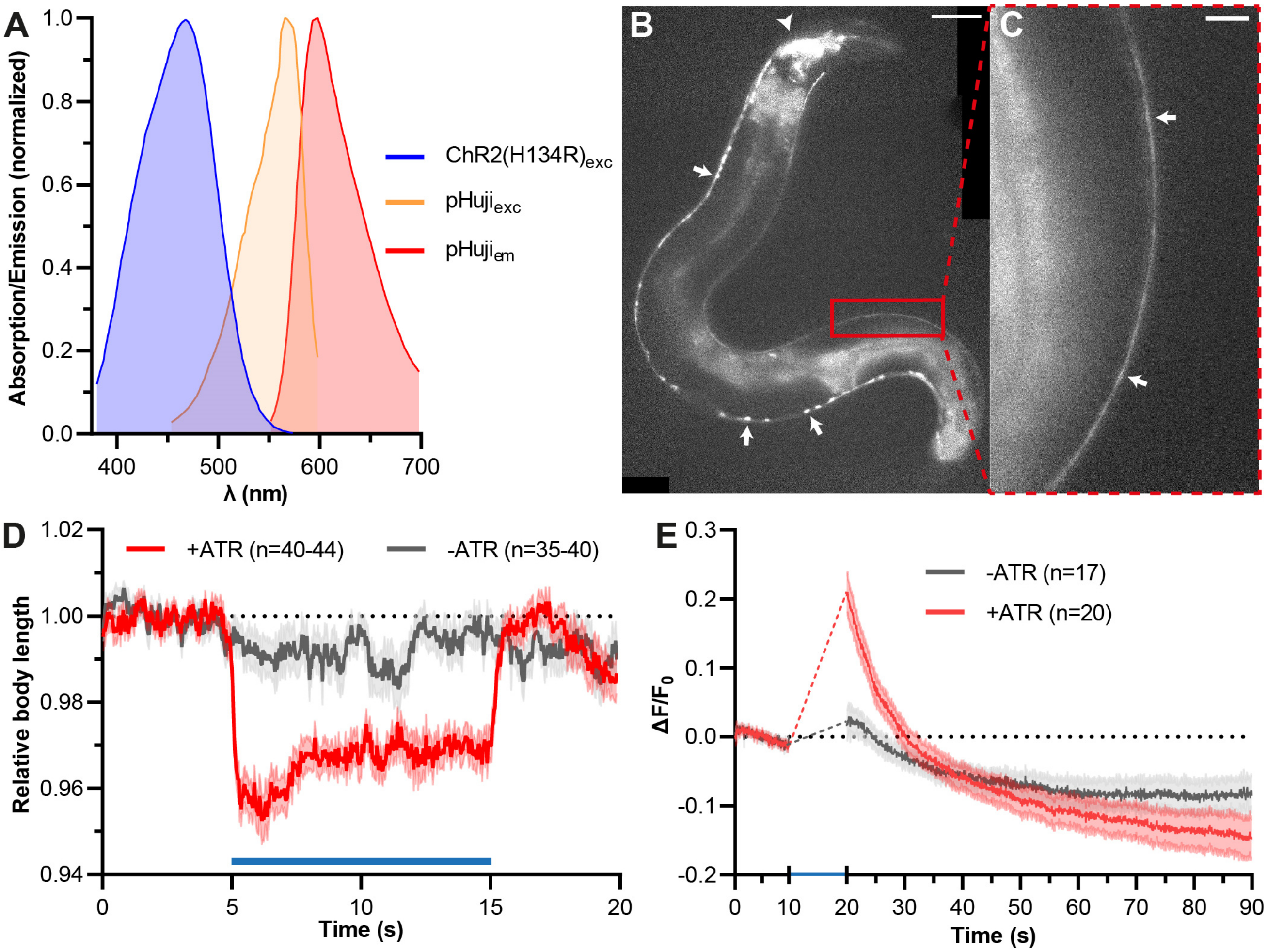

Fig. S1. Depolarization of cholinergic motor neurons with ChR2 triggers fusion of SVs containing SNG-1::pHuji. (A) Relative excitation and emission spectra of ChR2 and pHuji, normalized to the maximum absorption/emission amplitude. **(B)** Representative image of *C. elegans* expressing SNG-1::pHuji in cholinergic neurons. Arrows: Ventral location of cell bodies of A- and B-type motor neurons. Arrowhead: cholinergic neurons within the head ganglia. 40 x magnification. Scale bar, 50 µm. **(C)** Enlarged view of the dorsal nerve cord (DNC) in (C). Arrows: fluorescent puncta, representing SV clouds and neuronal plasma membrane. Scale bar, 10 µm. **(D)** Mean relative body length (± SEM) of animals expressing ChR2 and pHuji, optionally treated with ATR (as indicated), normalized to the average body length before stimulation. A 10 s continuous light pulse (470 nm, 0.34 mW/mm^2) was applied after 5 s (indicated by the blue bar). Number of animals indicated (n), accumulated from N = 3 biological replicates. **(E)** Mean (± SEM) relative change of DNC fluorescence of animals treated with and without ATR, before and after a 10 s continuous light pulse (460 nm, 0.34 mW/mm^2), applied after 10 s (note, fluorescence of pHuji cannot be properly imaged during blue light pulse, due to photoswitching). Number of animals indicated (n), accumulated from N = 2 biological replicates.

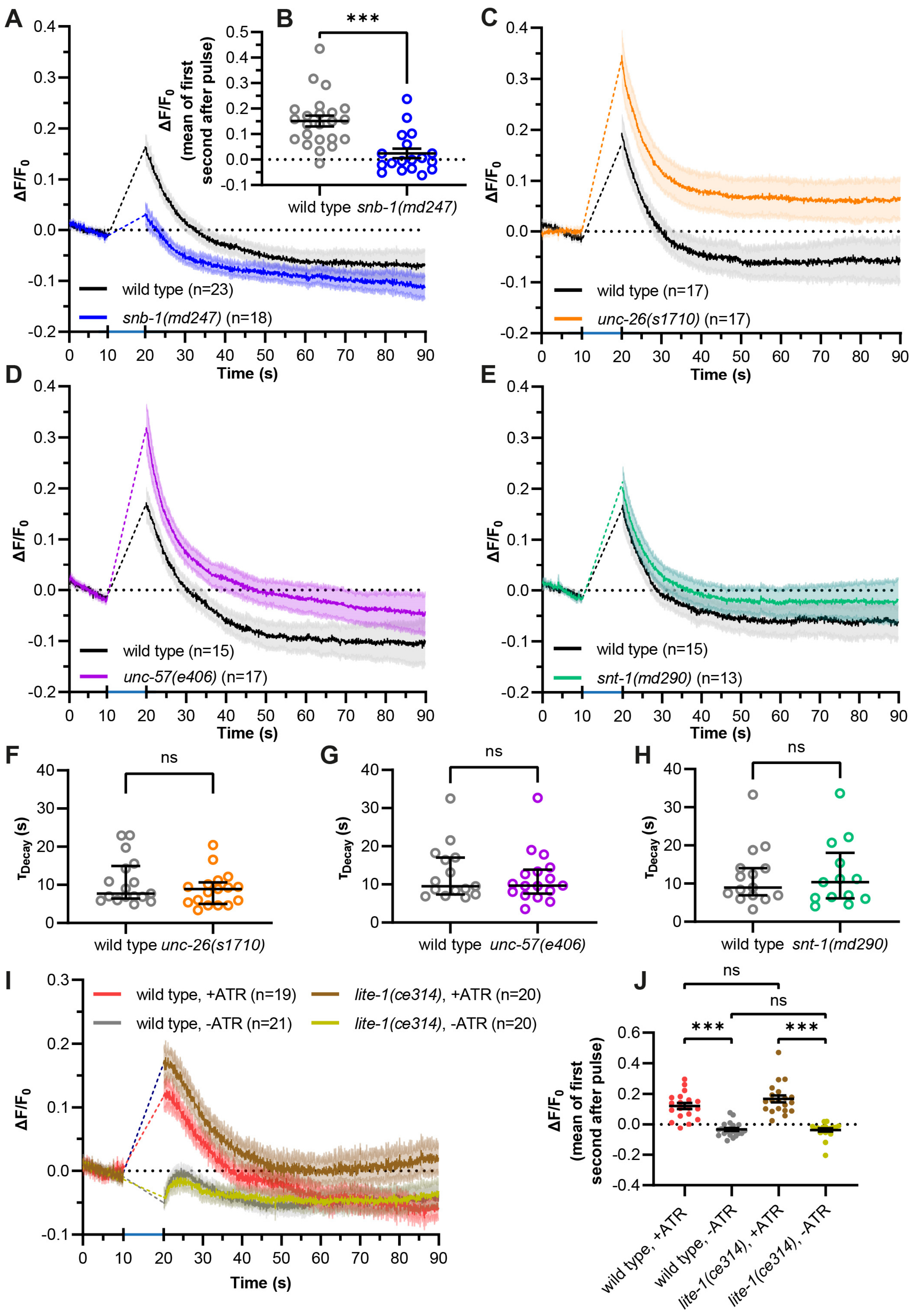
A
B
***
ΔF/F0 (mean of first second after pulse)
wild type
snb-1(md247)
ΔF/F0
wild type (n=23)
snb-1(md247) (n=18)
Time (s)
C
wild type (n=17)
unc-26(s1710) (n=17)
D
wild type (n=15)
unc-57(e406) (n=17)
E
wild type (n=15)
snt-1(md290) (n=13)
F
ns
τDecay (s)
wild type
unc-26(s1710)
G
ns
unc-57(e406)
H
ns
snt-1(md290)
I
wild type, +ATR (n=19)
wild type, -ATR (n=21)
lite-1(ce314), +ATR (n=20)
lite-1(ce314), -ATR (n=20)
J
ns
***
wild type, +ATR
wild type, -ATR
lite-1(ce314), +ATR
lite-1(ce314), -ATR

Fig. S2. pHuji fluorescence signal increase is affected by mutation of *snb-1(md247)* in pOpsicle assays. (A, C, D, E) Mean (± SEM) DNC fluorescence of wild type and mutant animals treated with ATR, and expressing ChR2 and SNG-1::pHuji in cholinergic motor neurons. A 10 s continuous light pulse (460 nm, 0.34 mW/mm^2, indicated by blue bar) was applied after 10 s. Number of animals is indicated (n), accumulated from N = 3 biological replicates. **(B)** Fluorescent signal of individual wild type and *snb-1(md247)* animals, as analyzed in (A), immediately following the end of the stimulation (20 – 21 s). Mean (± SEM). Unpaired *t*-Test; statistically significant difference is indicated as ***p < 0.001. **(F - H)** Calculated fluorescence decay constants of single animals using a one-phase exponential fit after stimulation (20 – 90 s). Median with interquartile range. Mann-Whitney test (ns, not significant, p > 0.05). In C – H, only animals showing a decay of fluorescence after stimulation were taken into consideration (wild type: 47 of 47 animals, *unc-26(s1710)*: 17 of 18, *unc-57(e406)*: 17 of 17, *snt-1(md290)*: 13 of 13). **(I)** Mean (± SEM) relative change of DNC fluorescence of wildtype and *lite-1(ce314)* animals treated with and without ATR, before and after a 10 s continuous light pulse (460 nm, 0.34 mW/mm^2), applied after 10 s. Number of animals indicated (n), accumulated from N = 3 biological replicates. **(J)** Fluorescent signal of individual wild type and *lite-1(ce314)* animals, as analyzed in (I), following the end of the stimulation (20 – 21 s). Mean (± SEM). One-way ANOVA with Tukey's correction (ns p > 0.05, ***p < 0.001).

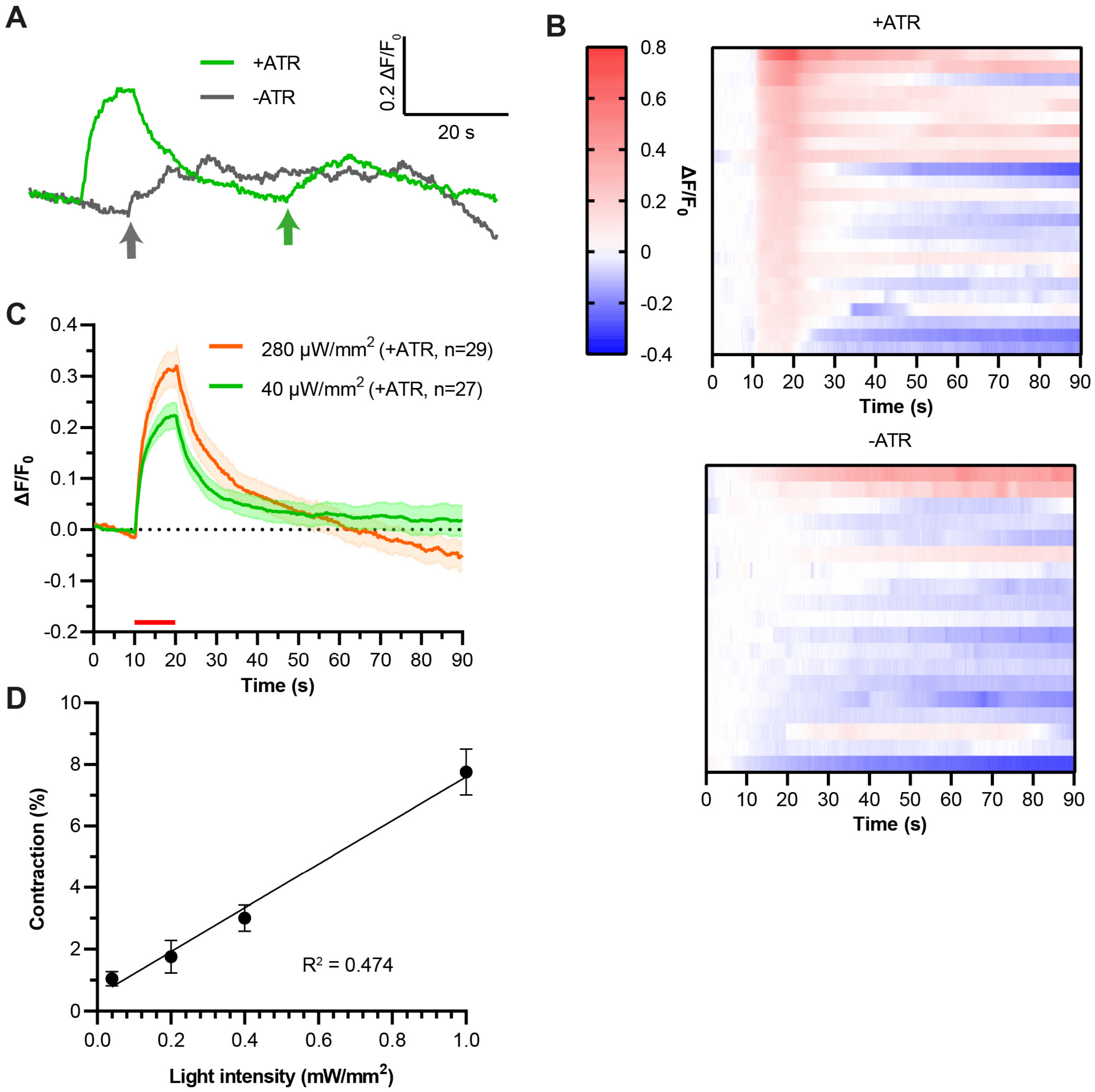
A
+ATR
-ATR
0.2 ΔF/F0
20 s
B
+ATR
0.8
0.6
0.4
0.2
0
-0.2
-0.4
ΔF/F0
0 10 20 30 40 50 60 70 80 90
Time (s)
-ATR
0 10 20 30 40 50 60 70 80 90
Time (s)
C
280 μW/mm2 (+ATR, n=29)
40 μW/mm2 (+ATR, n=27)
ΔF/F0
0.4
0.3
0.2
0.1
0.0
-0.1
-0.2
0 10 20 30 40 50 60 70 80 90
Time (s)
D
Contraction (%)
10
8
6
4
2
0
R2 = 0.474
0.0 0.2 0.4 0.6 0.8 1.0
Light intensity (mW/mm2)

Figure S3. pHluorin imaging. (A) Exemplary pHluorin fluorescence traces recorded from animals with or without ATR, exhibiting spontaneous signal increases (indicated by arrows). A 10 s continuous light pulse (590 nm, 40 µW/mm^2) was applied after 10 s. **(B)** Color-coded traces representing normalized DNC fluorescence of individual animals represented in Figure 2I in the pOpsicle assay. **(C)** Mean (± SEM) change of fluorescence of SNG-1::pHluorin co-expressed with ChrimsonSA in cholinergic motor neurons. A 10 s continuous light pulse (590 nm; indicated by a red bar) was applied after 10 s. Light intensity as indicated. Number of animals (n), accumulated from N = 4 (40 µW/mm^2), and N = 3 (280 µW/mm^2) biological replicates. **(D)** Mean relative contraction (± SEM) of animals expressing ChrimsonSA and pHluorin with ATR, as indicated, normalized to the average body length before stimulation. A 10 s continuous light pulse (580 nm) was applied as represented in Fig. 2D. Mean of seconds 6 – 14. 0.04 mW/mm^2 n = 41, 0.2 mW/mm^2 n = 29, 0.4 mW/mm^2 n = 33, 1 mW/mm^2 n= 29 accumulated from N = 2 - 3 biological replicates.

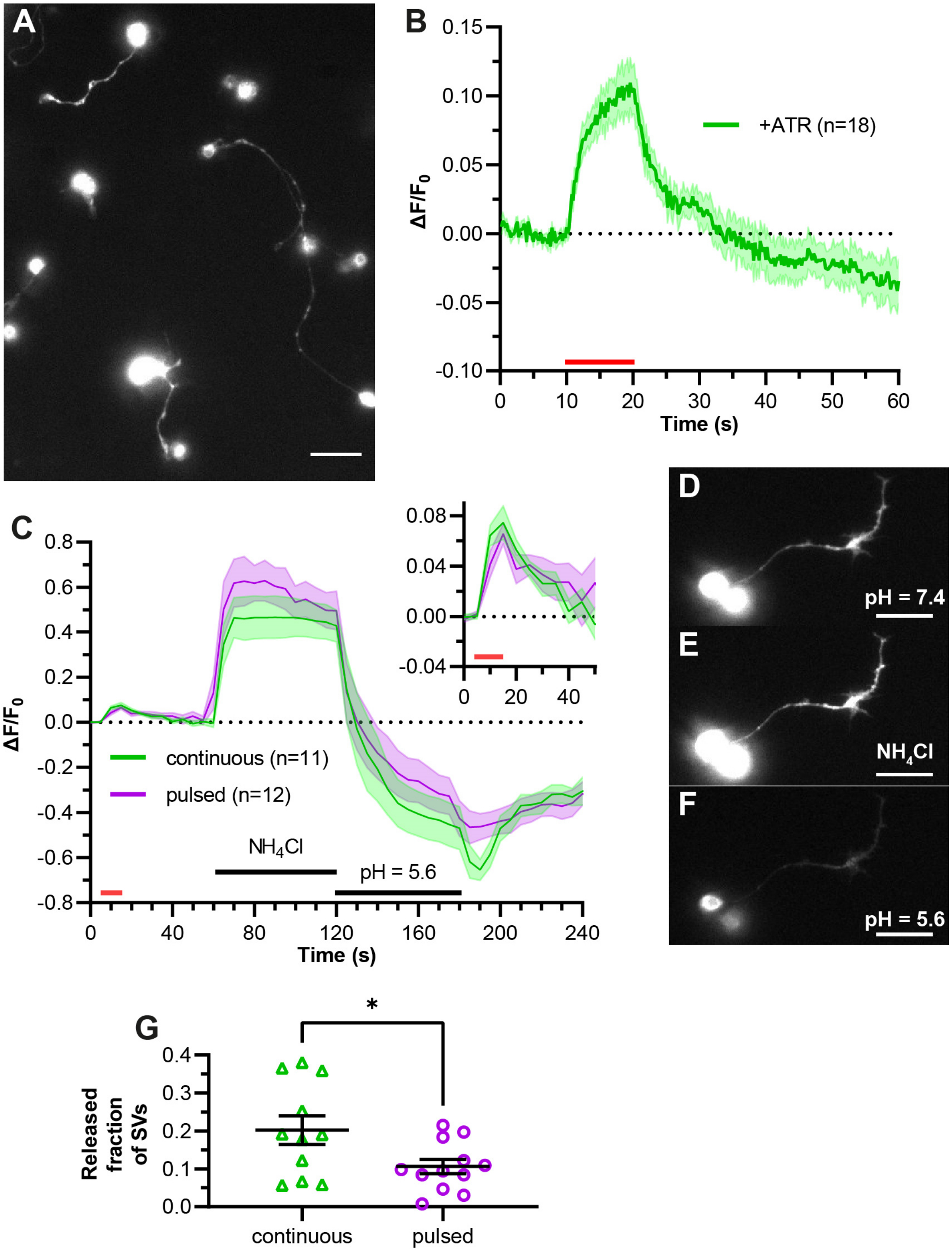
A
B
0.15
0.10
0.05
0.00
-0.05
-0.10
ΔF/F0
+ATR (n=18)
0
10
20
30
40
50
60
Time (s)
C
0.8
0.6
0.4
0.2
0.0
-0.2
-0.4
-0.6
-0.8
ΔF/F0
continuous (n=11)
pulsed (n=12)
NH4Cl
pH = 5.6
0
40
80
120
160
200
240
Time (s)
0.08
0.04
0.00
-0.04
0
20
40
D
pH = 7.4
E
NH4Cl
F
pH = 5.6
G
*
0.4
0.3
0.2
0.1
0.0
Released fraction of SVs
continuous
pulsed

Figure S4. Green pOpsicle in cultured primary cholinergic motor neurons (A) Representative image of primary neuronal cells expressing SNG-1::pHluorin. Scale bar, 10 µm. **(B)** Mean (± SEM) normalized neurite fluorescence of pHluorin expressing cells in primary neuronal cell culture that have been supplemented with ATR. A 10 s continuous light pulse (590 nm, 40 µW/mm^2) was applied after 10 s. Only cells showing a strong response during stimulation were taken into consideration (18 of 52), from N = 4 biological replicates. **(C)** Mean (± SEM) normalized neurite fluorescence of pHluorin expressing cells in primary neuronal cell cultures that have been supplemented with ATR. 0.2 frames per second. A 10 s continuous or 2 Hz (100 ms pulses) light stimulation (590 nm, 40 µW/mm^2) was applied after the second frame. NH_4Cl containing solution (HEPES buffered, pH = 7.4) was added after 60 s. MES buffered solution (pH = 5.6) was added after 120 s. Cells were washed three times with control saline buffer (180 s, HEPES buffered, pH = 7.4) before start of a new acquisition. Top right corner: zoom of 0 – 50 s. Number of measured neurons is indicated (n), from N = 3 biological replicates. Only cells showing a strong response during stimulation were taken into consideration (continuous: 11 of 52, pulsed: 12 of 35). **(D, E, F)** Representative pHluorin expressing neuron, treated with different buffers as indicated above scale bars. Scale bar, 10 µm. **(G)** Mean (± SEM) released fraction of SVs, calculated from measurements represented in (E). Each dot represents a single neuron. Unpaired *t*-Test (*p < 0.05).

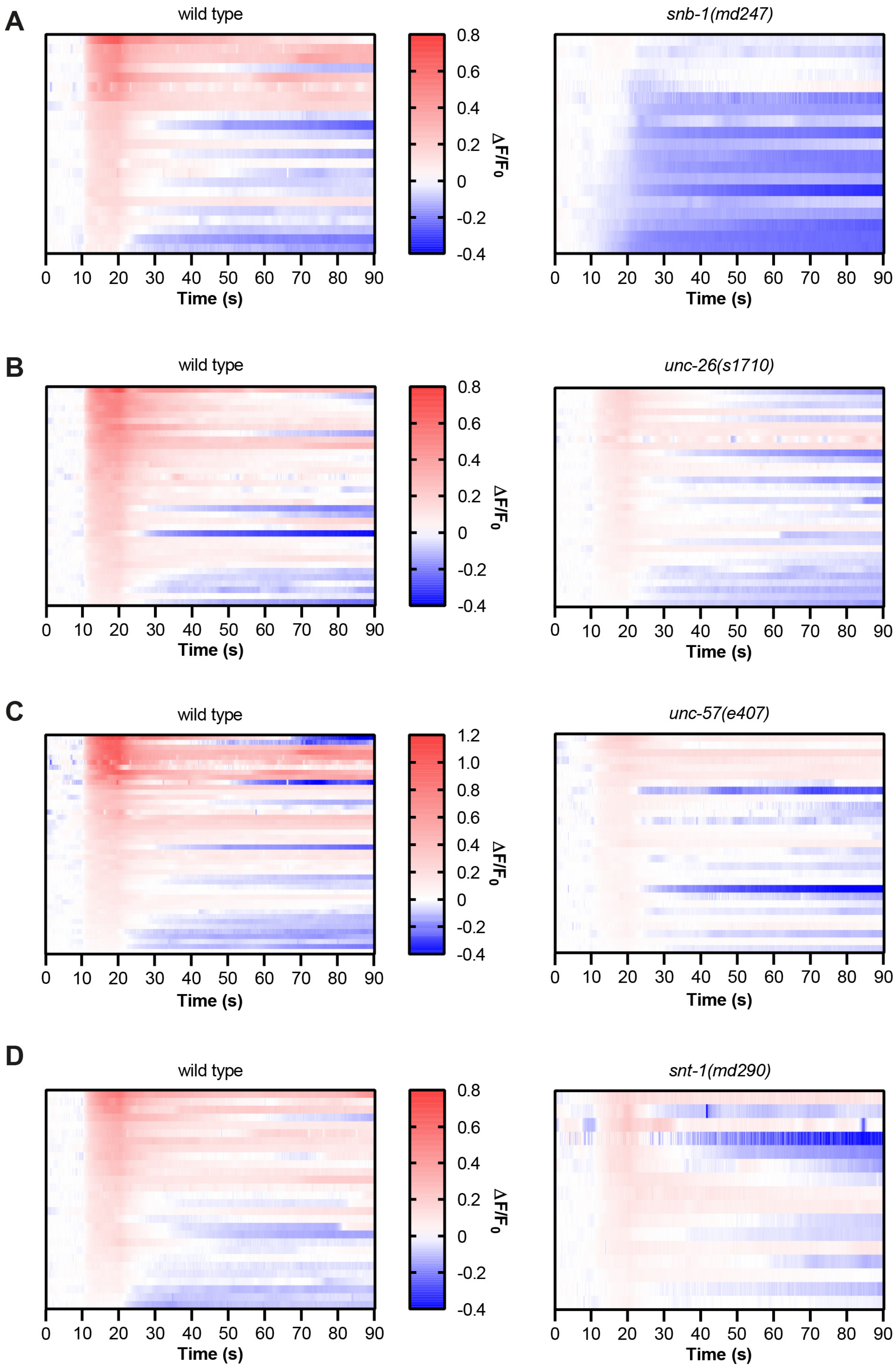
A
wild type
snb-1(md247)
B
wild type
unc-26(s1710)
C
wild type
unc-57(e407)
D
wild type
snt-1(md290)
Time (s)
ΔF/F0
0
10
20
30
40
50
60
70
80
90
0.8
0.6
0.4
0.2
0
-0.2
-0.4
1.2
1.0

Figure S5. Individual traces of wildtype and mutant animals analyzed with the 'green' pOpsicle using continuous stimulation. Color-coded traces representing normalized DNC fluorescence of individual animals depicted in Figures 3A, C, G and K in the pOpsicle assay.

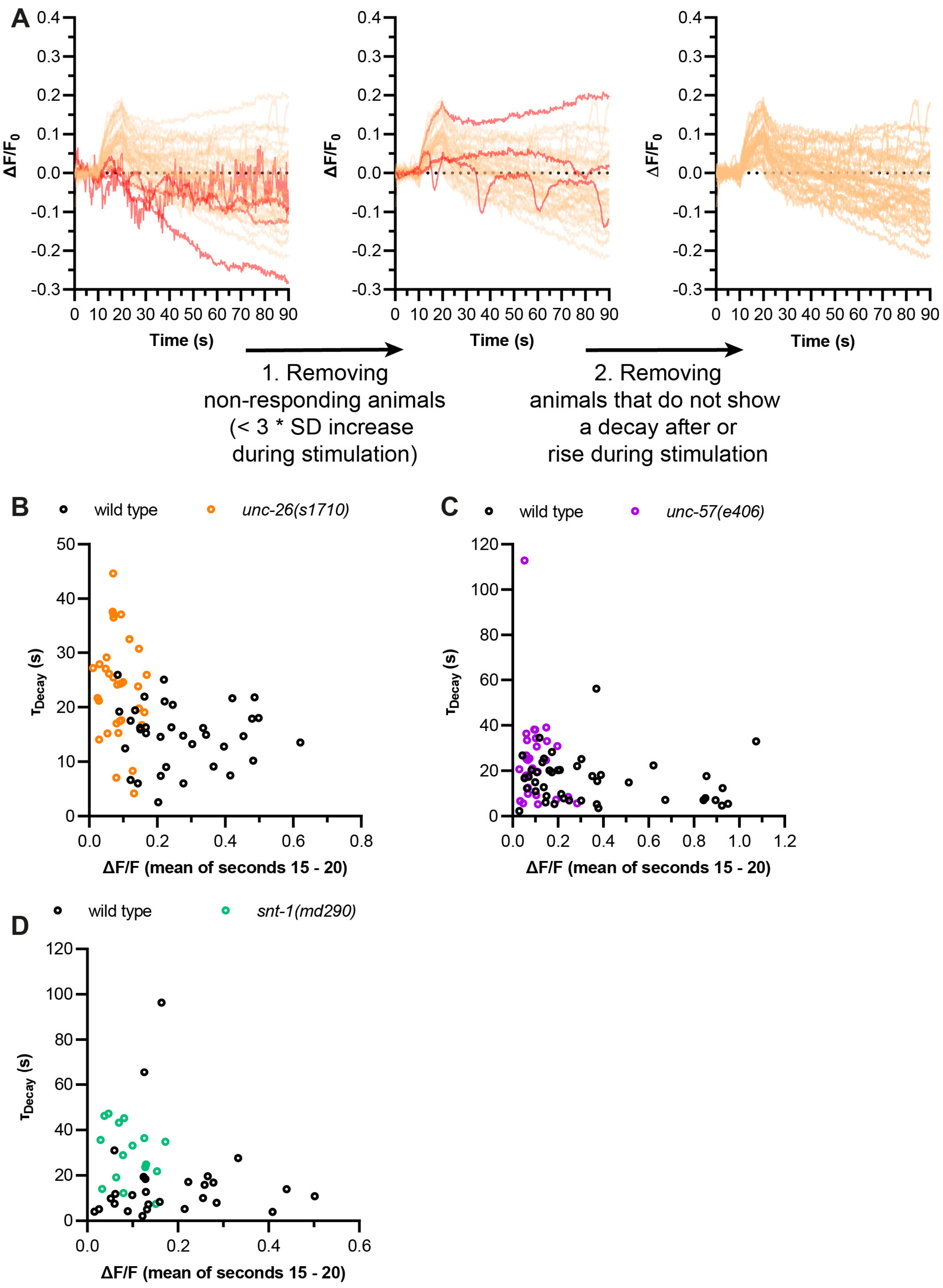
A
ΔF/F0
Time (s)
1. Removing non-responding animals (< 3 * SD increase during stimulation)
2. Removing animals that do not show a decay after or rise during stimulation
B
wild type
unc-26(s1710)
τDecay (s)
ΔF/F (mean of seconds 15 - 20)
C
wild type
unc-57(e406)
τDecay (s)
ΔF/F (mean of seconds 15 - 20)
D
wild type
snt-1(md290)
τDecay (s)
ΔF/F (mean of seconds 15 - 20)

Figure S6. Analysis of fluorescence decay time constants with the 'green' pOpsicle. (A) Traces of individual *unc-26(s1710)* mutant animals as represented in Figure 3C are shown as an example of which datasets were removed for the analysis of fluorescence decay time constants. Removed datasets in each step are shown in red. Step 1: The maximum background corrected fluorescence during stimulation was calculated (as a moving average of 1 s). The animal was removed from analysis if this was lower than the average background corrected fluorescence before the stimulation + 3 * standard deviations (SD) of background corrected fluorescence before stimulation. Step 2: One-phase exponential decay fits after stimulation were inspected. If they did not show a decrease, the respective animal was discarded. Similarly, if the one-phase exponential rise fit during stimulation did not show an increase, the animal was disregarded in the analysis. **(B - D)** Fluorescence signals of individual wild type and mutant animals at the end of continuous stimulation (15 – 20 s) were compared to the respective calculated fluorescence decay constants. No significant correlation could be found for any of the datasets ($p > 0.05$). Spearman correlation was used.

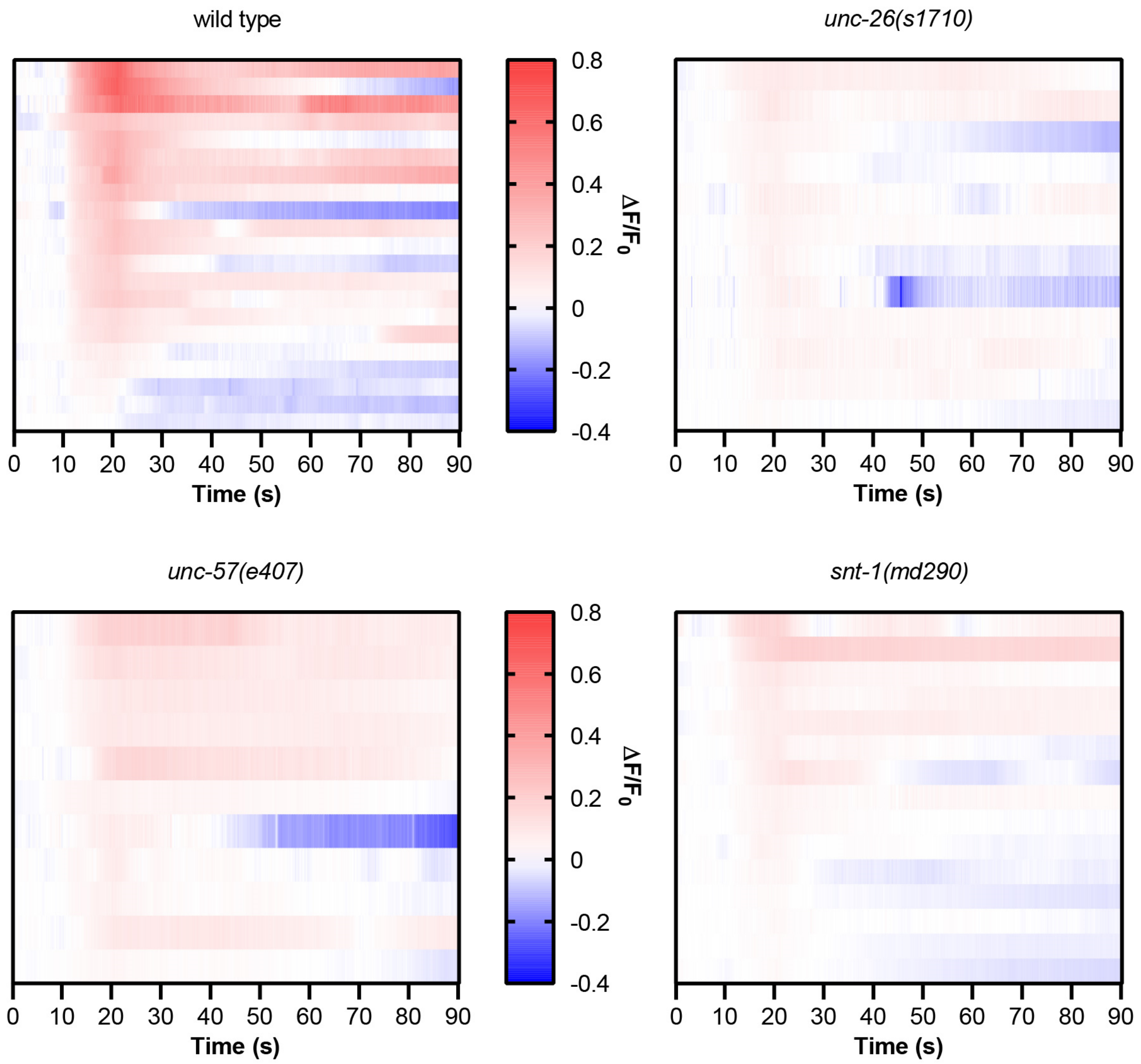

Figure S7. Individual traces of wildtype and mutant animals analyzed with the 'green' pOpsicle using pulsed stimulation. Color-coded traces representing normalized DNC fluorescence of individual animals depicted in Figure 4A in the pOpsicle assay.

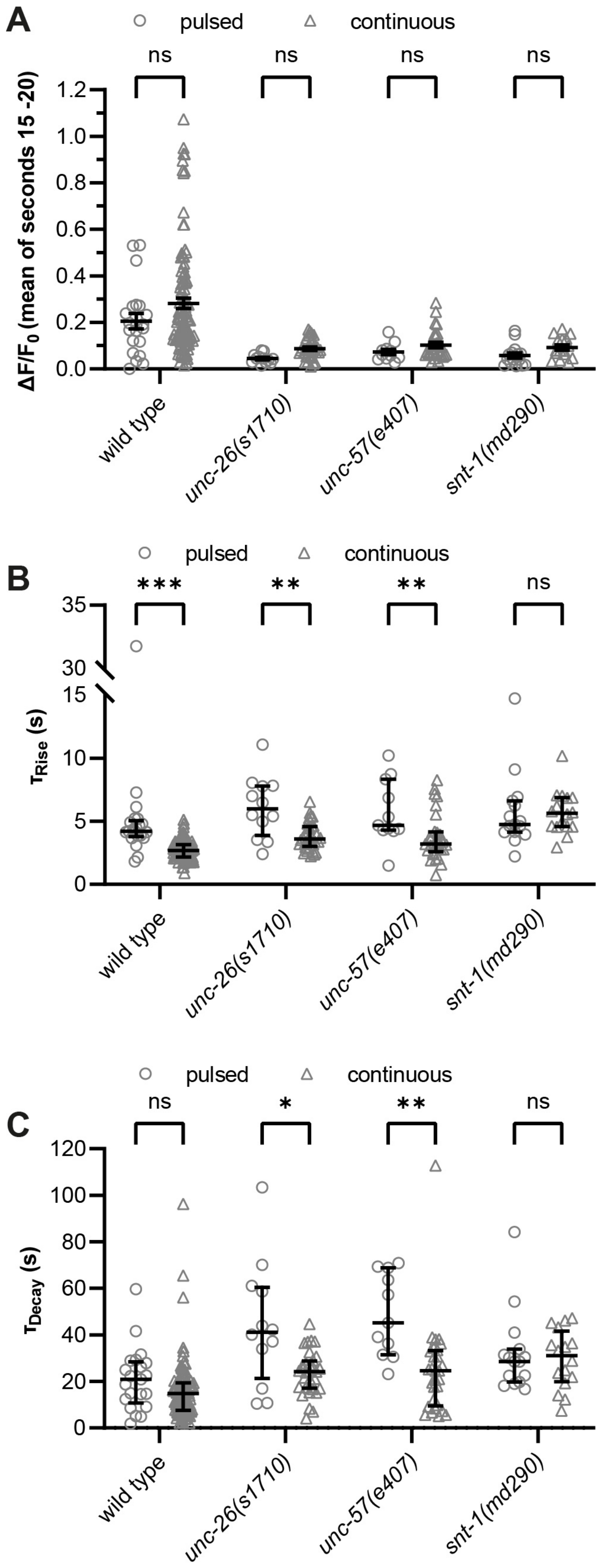
A
pulsed
continuous
ns
ns
ns
ns
ΔF/F0 (mean of seconds 15 -20)
1.2
1.0
0.8
0.6
0.4
0.2
0.0
wild type
unc-26(s1710)
unc-57(e407)
snt-1(md290)
B
pulsed
continuous
***
**
**
ns
35
30
15
10
5
0
τRise (s)
wild type
unc-26(s1710)
unc-57(e407)
snt-1(md290)
C
pulsed
continuous
ns
*
**
ns
120
100
80
60
40
20
0
τDecay (s)
wild type
unc-26(s1710)
unc-57(e407)
snt-1(md290)

Figure S8. Comparison between continuous and pulsed stimulation. (A) Fluorescent signal of individual wild type and mutant animals at the end of either pulsed or continuous stimulation (15 – 20 s). Mean (± SEM). Two-way ANOVA using Sidak's correction for multiple comparisons (ns, not significant). **(B)** Calculated fluorescence rise constants of single animals using a one-phase exponential fit during stimulation (10 – 20 s). Median with interquartile range. Multiple Mann-Whitney tests with Holm-Sidak correction for multiple comparisons (ns, not significant, $p > 0.05$, $^{**}p < 0.01$, $^{***}p < 0.001$). **(C)** Calculated fluorescence decay constants of single animals using a one-phase exponential fit after stimulation (20 – 90 s). Median with interquartile range. Mann-Whitney test (ns, not significant, $p > 0.05$, $^{*}p < 0.05$). Continuous: wild type n = 107 (pooled from animals depicted in Figure 3C – N), *unc-26(s1710)* n = 32, *unc-57(e407)* n = 29, *snt-1(md290)* n = 16. Pulsed: wild type n = 21, *unc-26(s1710)* n = 12, *unc-57(e407)* n = 11, *snt-1(md290)* n = 15.

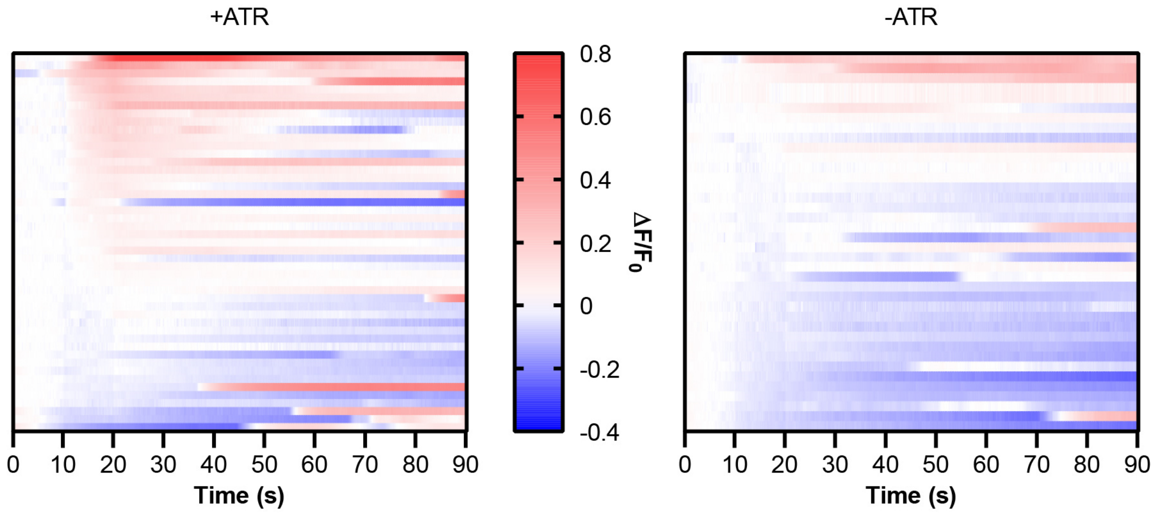

Figure S9. Individual traces of the green pOpsicle assay in RIM neurons. Color-coded traces representing normalized pHluorin fluorescence in RIM neurons of individual animals depicted in Figure 5F during the pOpsicle assay.
